# Supplementary material for: Global health effects of future atmospheric mercury emissions
Source: Nat Commun. 2021 May 24;12:3035. doi: 10.1038/s41467-021-23391-7 (PMC8144432; doi:10.1038/s41467-021-23391-7)
Supplement: Supplementary file 1 — Supplementary Information [file 41467_2021_23391_MOESM1_ESM.pdf]

Supplementary Materials for

Global Health Effects of Future Atmospheric Mercury Emissions

Yanxu Zhang[1,*], Zhengcheng Song[1], Shaojian Huang[1], Peng Zhang[1], Yiming Peng[1], Peipei Wu[1], Jing Gu[1], Stephanie Dutkiewicz[2], Huanxin Zhang[3,4], Shiliang Wu[4,5], Feiyue Wang[6], Long Chen[7], Shuxiao Wang[8,9], Ping Li[10]

[1]School of Atmospheric Sciences, Nanjing University, Nanjing, 210023, China

[2]Department of Earth, Atmospheric and Planetary Sciences, Massachusetts Institute of Technology, Cambridge, Massachusetts, USA

[3]Department of Chemical and Biochemical Engineering, University of Iowa, Iowa City, Iowa, USA

[4]Geological and Mining Engineering and Sciences, Michigan Technological University, Houghton, Michigan, USA

[5]Civil and Environmental Engineering, Michigan Technological University, Houghton, Michigan, USA

[6]Centre for Earth Observation Science, Department of Environment and Geography, University of Manitoba, Winnipeg, MB R3T 2N2, Canada

[7]Key Laboratory of Geographic Information Science (Ministry of Education), School of Geographic Sciences, East China Normal University, Shanghai 200241, China

[8]School of Environment, State Key Joint Laboratory of Environment Simulation and Pollution Control, Tsinghua University, Beijing 100084, P. R. China

[9]State Environmental Protection Key Laboratory of Sources and Control of Air Pollution Complex, Beijing 100084, P. R. China

[10]State Key Laboratory of Environmental Geochemistry, Institute of Geochemistry, Chinese Academy of Sciences, Guiyang, China

*Correspondence to: Yanxu Zhang, zhangyx@nju.edu.cn

Model Evaluation

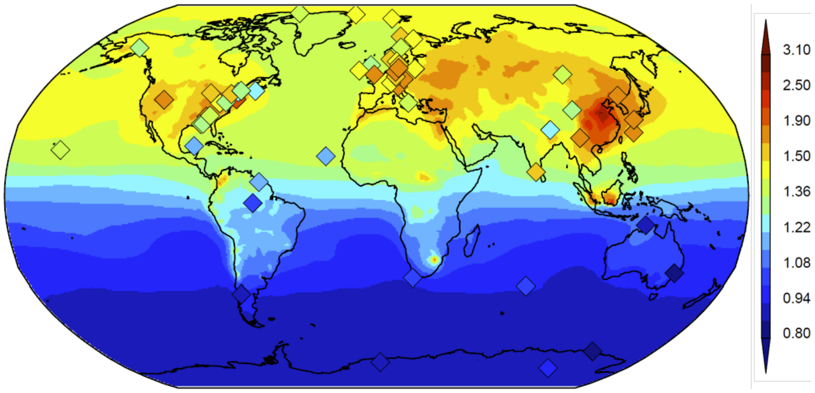

Fig. S1. Comparison of modeled annual mean atmospheric total gaseous Hg concentrations (background) in the surface air against observations (symbols). Data are as previously compiled by Horowitz et al.[1], Song et al.[2] and Zhang et al.[3]. Note the change in the linear color scale at 1.50 ng m^{-3}.

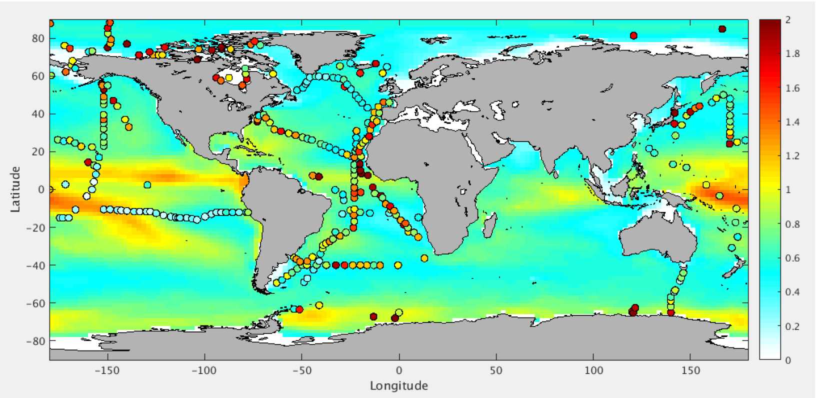

Fig. S2. Comparison of modeled annual mean surface ocean total Hg concentrations (pM, background) against observations (symbols). Data are as previously compiled by Bowman et al.[4] and Zhang et al.[5].

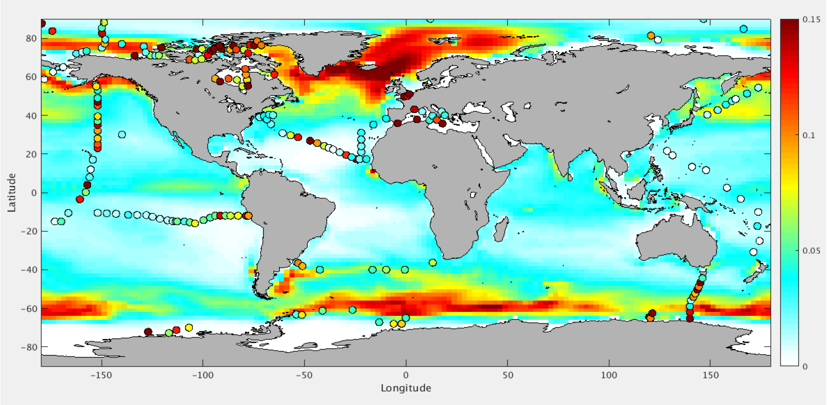

Fig. S3. Comparison of modeled annual mean surface ocean total MeHg concentrations (pM, background) against observations (symbols). Data are as previously compiled by Bowman et al.[4] and Zhang et al.[6].

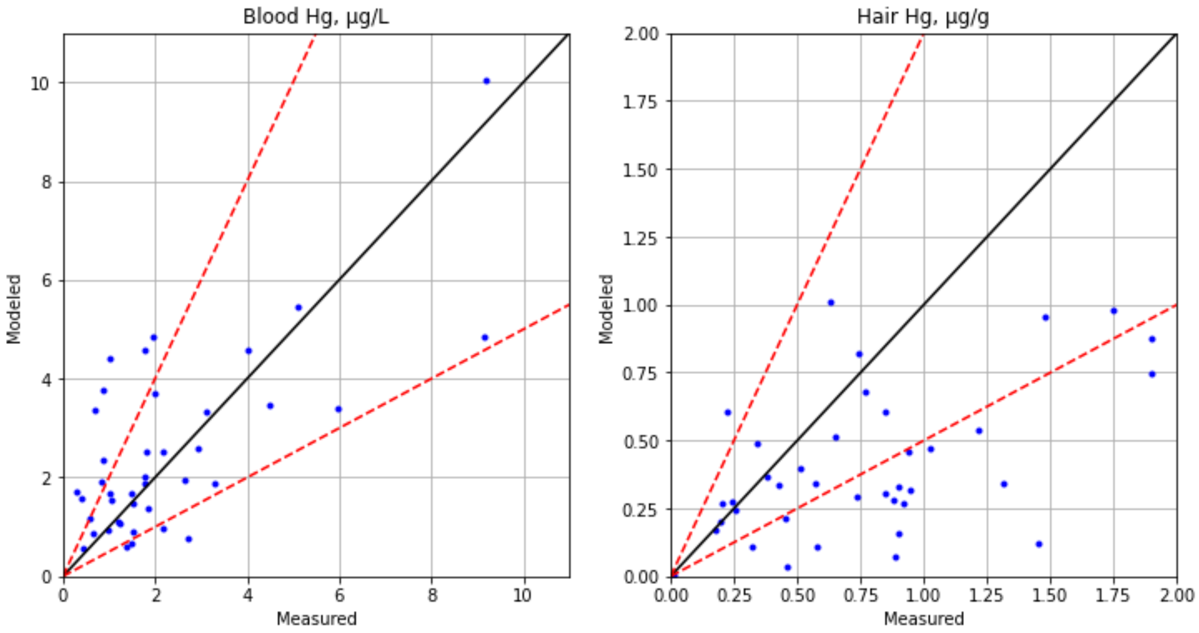

Fig. S4. Comparison between modeled and measured mercury biomarker concentrations for individual countries. Left) Blood Hg; Right) Hair Hg. Measured data are collected from literature (a detailed list of literature is provided below).

MeHg exposure

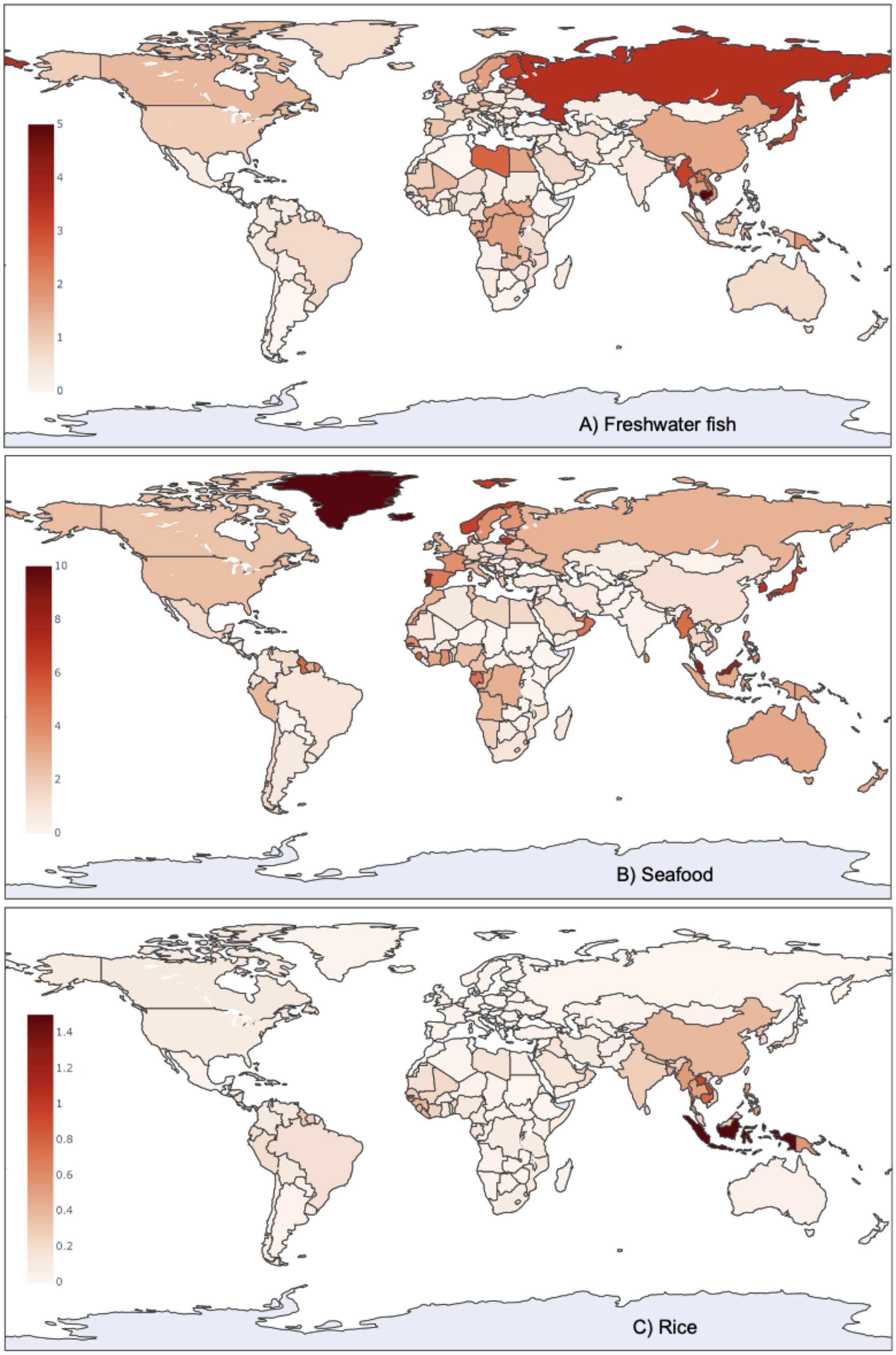

Fig. S5. MeHg exposure of general population in individual countries from the intake of different food: A) Freshwater fish/aquatic animals, B) Seafood, and C) Rice. Note that the color scales are in log-scale, and the ranges are different for the three categories of food to highlight the spatial patterns.

Model uncertainties

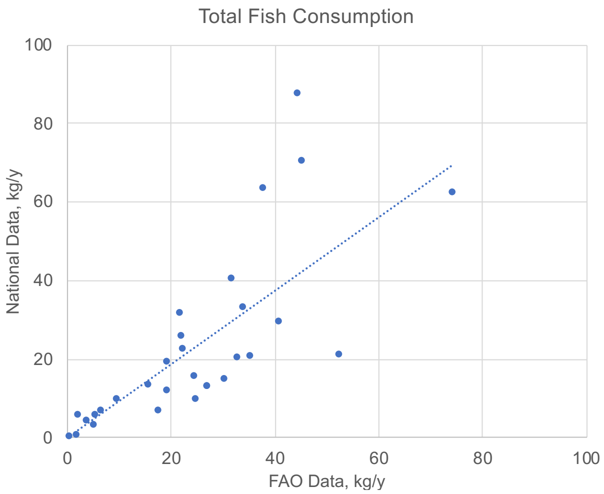

Fig. S6. Comparison between FAO and national data for total fish consumption of individual countries. The national data of 39 countries are collected from the literature. These countries include Tuvalu, Samoa, Niue Wallis and Futuna, French Polynesia, FSM, Kiribati, Nauru, Cook Island, Palau, Solomon Islands, New Caledonia, Fiji, Vanuatu, Tonga, Papua New Guinea, Cambodia, Philippines, Thailand, Myanmar, Lao PDR, Vietnam, Indonesia, Timor Leste, Sri Lanka, Bangladesh, India, Pakistan, China, Bhutan, Mongolia, Kenya, South Africa, Zambia, Australia, Brazil, Japan, Nigeria, and the United States. Data sources include household surveys in the Asia-Pacific region[7,8,17,9–16].

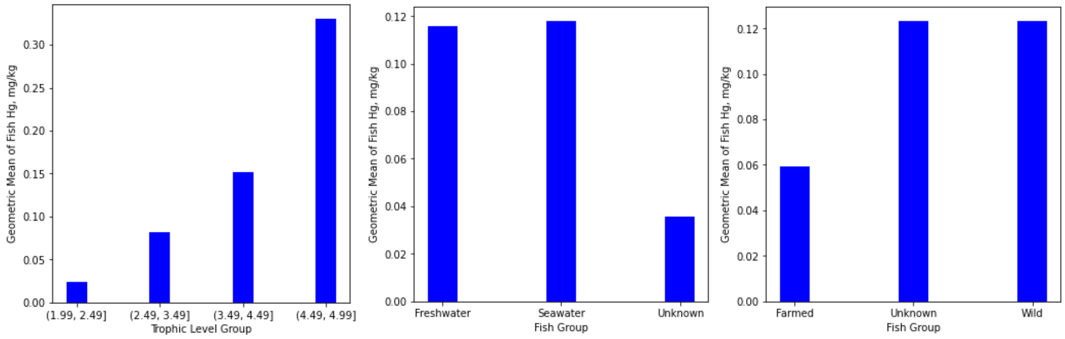

Fig. S7. MeHg levels in different groups of fish/aquatic animals (left: trophic level, middle: water type, right: farm raised or wild caught).

References

1. Horowitz, H. M. *et al.* A new mechanism for atmospheric mercury redox chemistry: Implications for the global mercury budget. *Atmos. Chem. Phys.* **17**, 6353–6371 (2017).

2. Song, S. *et al.* Top-down constraints on atmospheric mercury emissions and implications for global biogeochemical cycling. *Atmos. Chem. Phys.* **15**, 7103–7125 (2015).

3. Zhang, Y. *et al.* Observed decrease in atmospheric mercury explained by global decline in anthropogenic emissions. *Proc. Natl. Acad. Sci. U. S. A.* **113**, 526–531 (2016).

4. Bowman, K. L., Lamborg, C. H. & Agather, A. M. A global perspective on mercury cycling in the ocean. *Sci. Total Environ.* **710**, 136166 (2020).

5. Zhang, Y. *et al.* Biogeochemical drivers of the fate of riverine mercury discharged to the global and Arctic oceans. *Global Biogeochem. Cycles* **29**, 854–864 (2015).

6. Zhang, Y., Soerensen, A. L., Schartup, A. T. & Sunderland, E. M. A Global Model for Methylmercury Formation and Uptake at the Base of Marine Food Webs. *Global Biogeochem. Cycles* **34**, e2019GB006348 (2020).

7. Needham, S. & Funge-Smith, S. *The consumption of fish and fish products in the Asia-Pacific region based on household surveys*. *Food and Agriculture Organisation of the United Nations* (2015).

8. Farm Africa. Market study of the aquaculture market in Kenya. (2016). Available at: https://www.larive.com/case/farm-africanetherlands-embassy-to-kenya. (Accessed: 22nd April 2021)

9. Statistics South Africa. *Fishery Accounts for South Africa: 1990−2011*. (Statistics South Africa, 2013).

10. Tran, N. *et al.* Fish supply and demand for food security in Sub-Saharan Africa: An analysis of the Zambian fish sector. *Mar. Policy* **99**, 343–350 (2019).

11. Australian Bureau of Agricultural and Resource Economics and Sciences. Australian fisheries and aquaculture statistics 2016. *Fisheries Research and Development Corporation project 2014/245* (2017). Available at: https://www.agriculture.gov.au/sites/default/files/sitecollectiondocuments/abares/publications/AustFishAquacStats_2016_v1.0.0.pdf. (Accessed: 22nd April 2021)

12. Barone, R. S. C., Lorenz, E. K., Sonoda, D. Y. & Cyrino, J. E. P. Fish and fishery products trade in Brazil, 2005 to 2015: A review of available data and trends. *Sci. Agric.* **74**, 417–424 (2017).

13. Statistics Canada. Food available in Canada. (2020). Available at: https://www150.statcan.gc.ca/t1/tbl1/en/tv.action?pid=3210005401. (Accessed: 22nd April 2021)

14. Poblete, E. G., Drakeford, B. M., Ferreira, F. H., Barraza, M. G. & Failler, P. The impact of trade and markets on Chilean Atlantic salmon farming. *Aquac. Int.* **27**, 1465–1483 (2019).

15. EUMOFA. European Market Observation for Fisheries and Aquaculture Products. (2020). Available at:

https://data.europa.eu/data/datasets/european-market-observatory-for-fisheries-and-aquaculture-products?locale=en. (Accessed: 22nd April 2021)

16. Statistics Japan. Japan Statistical Yearbook 2020. (2020). Available at: https://www.stat.go.jp/english/data/nenkan/69nenkan/index.html. (Accessed: 22nd April 2021)

17. WorldFish. WorldFish in Nigeria: Factsheet. (2017). Available at: https://digitalarchive.worldfishcenter.org/bitstream/handle/20.500.12348/267/4132_2017-17.pdf. (Accessed: 22nd April 2021)

Literature list for food MeHg concentrations

Fish/aquatic animals

1. Ababneh FA, Al-Mamani IF, 2012. Levels of mercury, cadmium, lead and other selected elements in canned tuna fish commercialised in Jordan. Int J Environ Anal Chem, DOI:10.1080/03067319.2012.672981.
2. Abolghait SK, Garbaj AM, 2015. Determination of cadmium, lead and mercury residual levels in meat of canned light tuna (Katsuwonus pelamis and Thunnus albacares) and fresh little tunny (Euthynnus alletteratus) in Libya, open veterinary Journal, 2015, 5, 130-137.
3. Acosta-Lizarraga, L. G., Berges-Tiznado, M. E., Bojorquez-Sanchez, C., Osuna-Martinez, C. C., Paez-Osuna, F., 2020. Bioaccumulation of mercury and selenium in tissues of the mesopelagic fish Pacific hake (Merluccius productus) from the northern Gulf of California and the risk assessment on human health. Chemosphere, 225, 126941.
4. Adams D, Sonneb C, Basuc N, Dietzb R, Namc D, Leifssond P, et al. 2010. Mercury contamination in spotted seatrout, Cynoscion nebulosus: An assessment of liver, kidney, blood, and nervous system health. Sci Total Environ 408(23):5808-5816.
5. Adams DH, McMichael RH. 1999. Mercury levels in four species of sharks from the Atlantic coast of Florida. Fishery Bulletin 97, 372-379.
6. Adams DH, McMichael RH. 2007. Mercury in king mackerel, Scomberomorus cavalla, and Spanish mackerel, S-maculatus, from waters of the south-eastern USA: regional and historical trends. Mar Freshw Res 58(2):187-193.
7. Adams DH. 2004. Total mercury levels in tunas from offshore waters of the Florida Atlantic coast. Mar Pollut Bull 49, 659-663.
8. Agorku E., Voegborlo R.B., Adimado A., 2009. Total mercury levels in nice species of freshwater fish from two hydroelectric reservoirs and a crater lake in Ghana. Environmental monitoring and assessment, 153, 383–389.
9. Akerblom, S., Nilsson, M., Yu, J., Ranneby, B., Johansson, K., 2012. Temporal change estimation of mercury concentrations in northern Pike (Esox lucius L.) in Swedish lakes. Chemosphere, 86, 439-445.
10. Al-Abyadh, MKO, Babaqi, A.S., Al-Abyadh, M.M., Al-kaf, A.G., 2018. The Effects of Lead, Cadmium, Mercury and Arsenic on Fish and Seawater in Red Sea and the Gulf of Aden at Three Different Locations in Yemen. SF J Pharm Anal Chem. 1, 1002.
11. Andersen JL, Depledge MH. 1997. A survey of total mercury and methylmercury in edible fish and invertebrates from Azorean waters. Mar Environ Res 44(3):331-350.
12. Angyus, S.Bogdan., Darvasi, Eugen., Ponta, Michaela., Petreus, Dorin., Etz, Radu., Senila, Marin., Frentiu, Maria., Frentiu, Tiberiu., 2020. Interference-free, green microanalytical method for total mercury and methylmercury determination in biological and environmental samples using small-sized electrothermal vaporization capacitively coupled plasma microtorch optical emission spectrometry. Talanta, 217, 121067.
13. Ashraf W. 2006. Levels of selected heavy metals in tuna fish. Arab J Sci Eng 31(1A):89-92.
14. Augelli MA, Munoz RAA, Richter EM, Cantagallo MI, Angnes L. 2007. Analytical procedure for total mercury determination in fishes and shrimps by chronopotentiometric stripping analysis at gold film electrodes after microwave digestion. Food Chem 101(2):579-584.

15. Baeyens W, Leermakers M, Papina T, Saprykin A, Brion N, Noyen J, et al. 2003. Bioconcentration and biomagnification of mercury and methylmercury in North Sea and Scheldt estuary fish. Arch Environ Contam Toxicol 45(4):498-508.
16. Balshaw S, Edwards JW, Ross KE, Ellis D, Padula DJ, Daughtry BJ. 2008. Empirical models to identify mechanisms driving reductions in tissue mercury concentration during culture of farmed southern bluefin tuna Thunnnus maccoyii. Mar Pollut Bull 56(12), 2009-2017.
17. Banana AAS, Mohamed RMSR, Al-Gheethi AAS. 2016. Mercury pollution for marine environmentat Farwa Island, Libya. J Environ Health Sci Eng. 14, 5.
18. Bank MS, Chesney E, Shine JP, Maage A, Senn DB. 2007. Mercury bioaccumulation and trophic transfer in sympatric snapper species from the Gulf of Mexico. Ecological Applications 17:2100-2110.
19. Barska I, Skrzynski I. 2003. Contents of methylmercury and total mercury in Baltic Sea fish and fish products. Bulletin of the Sea Fisheries Institute 3(160), 3-15.
20. Berntssen MHG, Julshamn K, Lundebye AK. 2010. Chemical contaminants in aquafeeds and Atlantic salmon (Salmo salar) following the use of traditional- versus alternative feed ingredients. Chemosphere 78(6), 637-646.
21. Bethune C, Seierstad SL, Seljeflot I, Johansen O, Arnesen H, Meltzer HM, et al. 2006. Dietary intake of differently fed salmon: a preliminary study on contaminants. European Journal of Clinical Investigation 36(3), 193-201.
22. Black FJ, Bokhutlo T, Somoxa A, Maethamako, M, Modisaemang O, Kemosedile T, Cobb-Adams C, Mosepele K. Chimbari M, 2011. The tropical African mercury anomaly: lower than expected mercury concentrations in fish and human hair. The Science of the total environment, 409, 1967–1975.
23. Blanco S, Gonzalez JC, Vieites JM. 2008. Mercury, cadmium and lead levels in samples of the main traded fish and shellfish species in Galicia, Spain. Food Addit Contam Part B-Surveill 1(1), 15-21.
24. Bloom NS. 1992. On the chemical form of mercury in edible fish and marine invertebrate tissue Can J Fish Aquat Sci 49(5), 1010-1017.
25. Bordajandi LR, Gomez G, Abad E, Rivera J, Fernandez-Baston MD, Blasco J, et al. 2004. Survey of persistent organochlorine contaminants (PCBs, PCDD/Fs, and PAHs), heavy metals (Cu, Cd, Zn, Pb, and Hg), and arsenic in food samples from Huelva (Spain): Levels and health implications. J Agric Food Chem 52(4):992-1001.
26. Boscher A, Gobert S, Guignard C, Ziebel J, L'Hoste L, Gutleb AC, et al. 2010. Chemical contaminants in fish species from rivers in the North of Luxembourg: Potential impact on the Eurasian otter (Lutra lutra). Chemosphere 78(7):785-792.
27. Brambilla G., Abete, M.C., Binato, G., Chiaravalle, E., Cossu, M., Dellatte, E., Miniero, R., Orletti, R., Piras, P., Roncarati, A., Ubaldi, A., Chessa, G., 2013. Mercury occurrence in Italian seafood from the Mediterranean Sea and possible intake scenarios of the Italian coastal population. Regulatory Toxicology and Pharmacology, 65, 269–277.
28. Braune B, Muir D, DeMarch B, Gamberg M, Poole K, Currie R, et al. 1999. Spatial and temporal trends of contaminants in Canadian Arctic freshwater and terrestrial ecosystems: a review. Sci Total Environ 230(1-3), 145-207.
29. Brinkmann L, Rasmussen JB. 2010. High levels of mercury in biota of a new Prairie irrigation reservoir with a simplified food web in Southern Alberta, Canada. Hydrobiologia 641(1), 11-21.

30. Brockman JD, Sharp N, Ngwenyama RA, Shelnutt LD, McElroy JA. 2009. The concentration and variability of selenium and mercury measured in vacuum-packed tuna fish. J Radioanal Nucl Chem 282(1), 45-48.
31. Buck D, Evers D, Adams E, DiGangi J, Beeler B, Samanek J, Petrlik J, Turnquist M, Speranskaya O, Regan K, Johnson S., 2019. A global-scale assessment of fish mercury concentrations and the identification of biological hotspots. Science of the Total Environment. 687, 956-966.
32. Burger J, Gaines KF, Boring CS, Stephens WL, Snodgrass J, Dixon C, et al. 2002. Metal levels in fish from the Savannah River: Potential hazards to fish and other receptors. Environ Res 89(1), 85-97.
33. Burger J, Gaines KF, Boring CS, Stephens WL, Snodgrass J, Gochfeld M. 2001. Mercury and selenium in fish from the Savannah River: Species, trophic level, and locational differences. Environ Res 87(2), 108-118.
34. Burger J, Gochfeld M, Jeitner C, Burke S, Stamm T, Snigaroff R, et al. 2007b. Mercury levels and potential risk from subsistence foods from the Aleutians. Sci Total Environ 384(1-3), 93-105.
35. Burger J, Gochfeld M, Jeitner C, Burke S, Stamm T. 2007c. Metal levels in flathead sole (Hippoglossoides elassodon) and great sculpin (Myoxocephalus polyacanthocephalus) from Adak Island, Alaska: Potential risk to predators and fishermen. Environ Res 103(1), 62-69.
36. Burger J, Gochfeld M, Shukla T, Jeitner C, Burke S, Donio M, et al. 2007a. Heavy metals in Pacific Cod (Gadus macrocephalus) from the Aleutians: Location, age, size, and risk. J Toxicol Env Health Part A 70(22), 1897-1911.
37. Burger J, Gochfeld M. 2004. Mercury in canned tuna: white versus light and temporal variation. Environ Res 96(3), 239-249.
38. Burger J, Gochfeld M. 2005. Heavy metals in commercial fish in New Jersey. Environ Res 99(3), 403-412.
39. Burger J, Gochfeld M. 2006. Mercury in fish available in supermarkets in Illinois: Are there regional differences. Sci Total Environ 367(2-3), 1010-1016.
40. Burger J, Jeitner C, Donio M, Shukla S, Gochfeld M. 2009. Factors Affecting Mercury and Selenium Levels in New Jersey Flatfish: Low Risk to Human Consumers. J Toxicol Env Health Part A 72(14), 853-860.
41. Burger J. 2009. Risk to consumers from mercury in bluefish (Pomatomus saltatrix) from New Jersey: Size, season and geographical effects. Environ Res 109(7), 803-811.
42. Bustamante P, Lahaye V, Durnez C, Churlaud C, Caurant F. 2006. Total and organic Hg concentrations in cephalopods from the North Eastern Atlantic waters: Influence of geographical origin and feeding ecology. Sci Total Environ 368(2-3), 585-596.
43. Butala SJM, Scanlan LP, Chaudhur SN, Perry DD, Taylor R. 2007. Interlaboratory bias in the determination of mercury concentrations in commercially available fish utilizing thermal decomposition/amalgamation atomic absorption spectrophotometry. J Food Prot 70(10), 2422-2425.
44. Cabanero AI, Carvalho C, Madrid Y, Batoreu C, Camara C. 2005. Quantification and speciation of mercury and selenium in fish samples of high consumption in Spain and Portugal. Biol Trace Elem Res 103(1), 17-35.
45. Cabanero AI, Madrid Y, Camara C. 2004. Selenium and mercury bioaccessibility in fish samples: an invitro digestion method. Anal Chim Acta 526(1), 51-61.
46. Cabanero AI, Madrid Y, Camara C. 2007. Mercury-selenium species ratio in representative fish samples and their bioaccessibility by an in vitro digestion method. Biol Trace Elem Res 119(3), 195-211.

47. Cai Y, Rooker JR, Gill GA, Turner JP. 2007. Bioaccumulation of mercury in pelagic fishes from the northern Gulf of Mexico. Can J Fish Aquat Sci 64(3), 458-469.
48. Caldwell RS, Buhler DR. 1983. Heavy metals in estuarine shellfish from Oregon Arch Environ Contam Toxicol 12(1), 15-23.
49. Campbell, L., Hecky, R.E., Dixon, D.G., Chapman, L.J., 2006. Food web structure and mercury transfer in two contrasting Ugandan highland crater lakes (East Africa). African Journal of Ecology, 44, 337–346.
50. Campbell, L., Osano, O., Hecky, R., Dixon, D., 2003b. Mercury in fish from three rift valley lakes (Turkana, Naivasha and Baringo), Kenya, East Africa. Environmental Pollution, 125, 281–286.
51. Campbell, L., Verburg, P., Dixon, D.G., Hecky, R.E., 2008. Mercury biomagnification in the food web of Lake Tanganyika (Tanzania, East Africa). The Science of the total environment, 402, 184–191.
52. Campbell, L.M., Balirwa, J., Dixon, D., Hecky, R., 2004. Biomagnification of mercury in fish from Thruston Bay, Napoleon Gulf, Lake Victoria (East Africa). African Journal of Aquatic Science, 29, 91–96.
53. Campbell, L.M., Hecky, R.E., Nyaundi, J., Muggide, R., Dixon, D.G., 2003a. Distribution and Food-web Transfer of Mercury in Napoleon and Winam Gulfs , Lake Victoria , East Africa. 29, 267–282.
54. Capelli R, Contardi V, Cosma B, Minganti V, Zanicchi G. 1983. A 4-year study on the distribution of some heavy metals in 5 marine organisms of the Ligurian Sea. Mar Chem 12(4), 281-293.
55. Capelli R, Drava G, Siccardi C, De Pellegrini R, Minganti V. 2004. Study of the distribution of trace elements in six species of marine organisms of the Ligurian Sea (North-Western Mediterranean) -Comparison with previous findings. Ann Chim 94(7-8), 533-546.
56. Capelli R, Minganti V, Bernhard M. 1987. Total mercury, organic mercury, copper, manganese, selenium, and zinc in Sarda sarda from the Guld of Genoa. Sci Total Environ 63, 83-99.
57. Cappon CJ. 1984. Content and chemical form of mercury and selenium in Lake Ontario salmon and trout. J Gt Lakes Res 10(4), 429-434.
58. Carbonell G, Bravo JC, Fernandez C, Tarazona JV. 2009. A New Method for Total Mercury and MethylMercury Analysis in Muscle of Seawater Fish. Bull Environ Contam Toxicol 83(2), 210-213.
59. Carvalho ML, Santiago S, Nunes ML. 2005. Assessment of the essential element and heavy metal content of edible fish muscle. Anal Bioanal Chem 382(2), 426-432.
60. Chen CY, Chen MH. 2003. Investigation of Zn, Cu, Cd and Hg concentrations in the oyster of Chi-ku, Tai-shi and Tapeng Bay, southwestern Taiwan. J Food Drug Anal 11(1):32-38.
61. Chen MH, Chen CY, Chang SK, Huang SW. 2007. Total and organic mercury concentrations in the white muscles of swordfish (Xiphias gladius) from the Indian and Atlantic oceans. Food Addit Contam 24(9), 969-975.
62. Cheung KC, Leung HM, Wong MH. 2008. Metal concentrations of common freshwater and marine fish from the Pearl River Delta, South China. Arch Environ Contam Toxicol 54(4), 705-715.
63. Chou C Chen CY, Chen MH. 2003. Investigation of Zn, Cu, Cd and Hg concentrations in the oyster of Chi-ku, Tai-shi and Tapeng Bay, southwestern Taiwan. J Food Drug Anal 11(1), 32-38.
64. Chouvelon T, Cresson P, Bouchoucha M, Brach-Papa C, Bustamante P, Crochet S, Marco-Mirallea F, Thomas B, Knoery J. 2018. Oligo-trophy as a major driver of mercury bioaccumulation in medium-to high-trophic level consumers: a marine ecosystem-comparative study. Environ Pollut 233, 844–854.
65. Chung SWC, Kwong KP, Tang ASP, Xiao Y, Ho PYY. 2008. Mercury and methylmercury levels in the main traded fish species in Hong Kong. Food Addit Contam Part B-Surveill 1(2), 106-113.

66. Ciardullo S, Aureli F, Coni E, Guandalini E, Lost F, Raggi A, et al. 2008. Bioaccumulation potential of dietary arsenic, cadmium, lead, mercury, and selenium in organs and tissues of rainbow trout (Oncorhyncus mykiss) as a function of fish growth. J Agric Food Chem 56(7), 2442-2451.
67. Cizdziel JV, Hinners TA, Heithmar EM. 2002. Determination of total mercury in fish tissues using combustion atomic absorption spectrometry with gold amalgamation. Water Air Soil Pollut 135(1-4), 355-370.
68. Collings SE, Johnson MS, Leah RT. 1996. Metal contamination of angler-caught fish from the Mersey Estuary. Mar Environ Res 41(3), 281-297.
69. Cross FA, Hardy LH, Jones NY, Barber RT. 1973. Relation between Total-Body Weight and Concentrations of Manganese, Iron, Copper, Zinc, and Mercury in White Muscle of Bluefish (Pomatomus saltatrix) and a Bathyl-Demersal Fish Antimora rostrata. Journal of the Fisheries Research Board of Canada 30(9), 1287-1291.
70. Cutshall NH, Naidu JR, Pearcy WG. 1978. Mercury concentrations in pacific hake, Merluccius productus (ayres), as a function of length and latitude. Science 200(4349), 1489-1491.
71. Dabeka R, McKenzie AD, Forsyth DS, Conacher HBS. 2004. Survey of total mercury in some edible fish and shellfish species collected in Canada in 2002. Food Addit Contam 21(5), 434-440.
72. Das YK, Aksoy A, Baskaya R, Duyar HA, Guvenc D, Boz V. 2009. Heavy Metal Levels of Some Marine Organisms Collected in Samsun and Sinop Coasts of Black Sea, in Turkey. Journal of Animal and Veterinary Advances 8(3), 496-499.
73. Davies IM. 2012. Department of Agriculture and Fisheries for Scotland (DAFS), Fish and Shellfish Landed at Scottish Ports (1975-1976). Aberdeen, Scotland.
74. De Marco SG, Botte SE, Marcovecchio JE. 2006. Mercury distribution in abiotic and biological compartments within several estuarine systems from Argentina: 1980-2005 period. Chemosphere, 65(2):213-223.
75. Del Gobbo LC, Archbold JA, Vanderlinden LD, Eckley CS, Diamond ML, Robson M. 2010. Risks and Benefits of Fish Consumption For Childbearing Women. Can J Diet Pract Res 71(1), 41-45.
76. Delaware River Basin Commission. 2011. Fish and Shellfish Tissue Data, DRBC EPA Coastal 2000. Available: http://www.state.nj.us/drbc/fishtiss.htm [accessed 3 February 2011].
77. Della Torre C, Petochi T, Corsi I, Dinardo MM, Baroni D, Alcaro L, et al. 2010. DNA damage, severe organ lesions and high muscle levels of As and Hg in two benthic fish species from a chemical warfare agent dumping site in the Mediterranean Sea. Sci Total Environ 408(9), 2136-2145.
78. Dellinger JA, Meyers RM, Gebhardt KJ, Hansen LK. 1996. The Ojibwa Health Study: Fish residue comparisons for Lakes Superior, Michigan, and Huron. Toxicol Ind Health 12(3-4), 393-402.
79. Deshpande A, Bhendigeri S, Shirsekar T, Dhaware D, Khandekar RN. 2009. Analysis of heavy metals in marine fish from Mumbai Docks. Environ Monit Assess 159(1-4), 493-500.
80. Deshpande AD, Draxler AFJ, Zdanowicz VS, Schrock ME, Paulson AJ. 2002. Contaminant Levels in Muscle of Four Species of Recreational Fish from the New York Bight Apex. NOAA Technical Memorandum NMFS-NE-157. NMFS-NE-157. http://www.nefsc.noaa.gov/publications/tm/tm157/tm157.pdf [accessed 24 February 2009].
81. Dewailly E, Ayotte P, Lucas M, Blanchet C. 2007. Risk and benefits from consuming salmon and trout: A Canadian perspective. Food Chem Toxicol 45(8), 1343-1348.

82. Dewailly E, Rouja P, Dallaire R, Pereg D, Tucker T, Ward J, et al. 2008. Balancing the risks and the benefits of local fish consumption in Bermuda. Food Addit Contam Part A-Chem 25(11), 1328-1338.
83. Domingo JL, Bocio A, Falco G, Llobet JM. 2007. Benefits and risks of fish consumption Part I. A quantitative analysis of the intake of omega-3 fatty acids and chemical contaminants. Toxicology 230(2-3), 219-226.
84. Doyon JF, Schetagne R, Verdon R. 1998. Different mercury bioaccumulation rates between sympatric populations of dwarf and normal lake whitefish (Coregonus clupeaformis) in the La Grande complex watershed, James Bay, Quebec. Biogeochemistry 40(2-3), 203-216.
85. Dsikowitzky, L., Mengesha, M., Dadebo, E., de Carvalho, C. E. V., Sindern, S., 2013. Assessment of heavy metals in water samples and tissues of edible fish species from Awassa and Koka Rift Valley Lakes, Ethiopia. Environmental monitoring and assessment, 185, 3117–3131.
86. Duarte FA, Bizzi CA, Antes FG, Dressler VL, Flores EMD. 2009. Organic, inorganic and total mercury determination in fish by chemical vapor generation with collection on a gold gauze and electrothermal atomic absorption spectrometry. Spectroc Acta Pt B-Atom Spectr 64(6), 513-519.
87. Eagles-Smith, C.A., Ackerman, J. T., Willacker, J.J., Tate, M.T., Lutz, M.A., Fleck, J A., Robin, Stewart, A., Wiener, J.G., Evers, D.C., Lepak, J.M., Davis, J.A., Pritz, C.F., 2016. patial and temporal patterns of mercury concentrations in freshwater fish across the Western United States and Canada. Science of The Total Environment, 568, 15, 1171-1184.
88. Easton MDL, Luszniak D, Von der Geest E. 2002. Preliminary examination of contaminant loadings in farmed salmon, wild salmon and commercial salmon feed. Chemosphere 46(7), 1053-1074.
89. Elhamri H, Idrissi L, Coquery M, Azemard S, El Abidi A, Benlemlih M, et al. 2007. Hair mercury levels in relation to fish consumption in a community of the Moroccan Mediterranean coast. Food Addit Contam 24(11), 1236-1246.
90. Elston R, Cake EW, Humphrey K, Isphording WC, Rensel JE. 2005. Dioxin and heavy-metal contamination of shellfish and sediments in St. Louis Bay, Mississippi and adjacent marine waters. J Shellfish Res 24(1), 227-241.
91. Endo T, Haraguchi K. 2010. High mercury levels in hair samples from residents of Taiji, a Japanese whaling town. Mar Pollut Bull 60(5), 743-747.
92. Endo, T., Haraguchi, K., Hotta, Y., Hisamichi, Y., Lavery, S., Dalebout, M.L., Baker, C.S., 2005. Total Mercury, Methyl Mercury, and Selenium Levels in the Red Meat of Small Cetaceans Sold for Human Consumption in Japan. Environmental Science & Technology, 39, 15, 5703–5708.
93. Escobar-Sanchez O, Galvan-Magana F, Rosiles-Martinez R. 2010. Mercury and Selenium Bioaccumulation in the Smooth Hammerhead Shark, Sphyrna zygaena Linnaeus, from the Mexican Pacific Ocean. Bull Environ Contam Toxicol 84(4), 488-491.
94. Ethier ALM, Scheuhammer AM, Bond DE. 2008. Correlates of mercury in fish from lakes near Clyde Forks, Ontario, Canada. Environ Pollut 154(1), 89-97.
95. Evans DW, Crumley PH. 2005. Mercury in Florida Bay fish: Spatial distribution of elevated concentrations and possible linkages to Everglades restoration. Bull Mar Sci 77(3), 321-345.
96. Fabris G, Turoczy NJ, Stagnitti F. 2006. Trace metal concentrations in edible tissue of snapper, flathead, lobster, and abalone from coastal waters of Victoria, Australia. Ecotox Environ Safe 63(2), 286-292.

97. Fairey R, Taberski K, Lamerdin S, Johnson E, Clark RP, Downing JW, et al. 1997. Organochlorines and other environmental contaminants in muscle tissues of sportfish collected from San Francisco Bay. Mar Pollut Bull 34(12), 1058-1071.
98. Falandysz J. 1990. Mercury content of squid Loligo opalescens. Food Chem 38(3), 171-177.
99. Ferreira AG, Faria VV, de Carvalho CEV, Lessa RPT, da Silva FMS. 2004. Total mercury in the night shark, Carcharhinus signatus in the western equatorial Atlantic Ocean. Braz Arch Biol Technol 47(4):629-634.
100. Fisher WS, Oliver LM, Winstead JT, Long ER. 2000. A survey of oysters Crassostrea virginica from Tampa Bay, Florida: associations of internal defense measurements with contaminant burdens. Aquat Toxicol 51(1), 115-138.
101. Food Standards Agency. 2003. Methylmercury in imported Fish and Shellfish and Their Products, UK Farmed Fish and Their Products. London. Available: http://www.food.gov.uk/multimedia/pdfs/fsis40_2003.pdf [accessed 14 October 2003].
102. Food Standards Agency. 2006. Survey of Metals and Other Elements in Processed Fish and Shellfish. London. Available: http://www.food.gov.uk/multimedia/pdfs/fsis0806.pdf [accessed May 3 2008].
103. Forsyth DS, Casey V, Dabeka RW, McKenzie A. 2004. Methylmercury levels in predatory fish species marketed in Canada. Food Addit Contam 21(9):849-856.
104. Freeman HC, Horne DA. 1973. Sampling edible muscle of swordfish (Xiphias gladius) for total mercury analysis. Journal of the Fisheries Research Board of Canada 30(8):1251-1252.
105. Freeman HC, Shum G, Uthe JF. 1978. Selenium content in swordfish (Xiphias gladius) in relation to total mercury content. J Environ Sci Health Part A-Environ Sci Eng Toxic Hazard Subst Control 13(3):235-240.
106. Garcia E, Carignan R. 2000. Mercury concentrations in northern pike (Esox lucius) from boreal lakes with logged, burned, or undisturbed catchments. Can J Fish Aquat Sci 57, 129-135.
107. Garcia-Hernandez J, Cadena-Cardenas L, Betancourt-Lozano M, Garcia-De-La-Parra LM, Garcia-Rico L, Marquez-Farias F. 2007. Total Mercury Content Found in Edible Tissues of Top Predator Fish From the Gulf of California, Mexico. Toxicological and Environmental Chemistry 89(3), 507-522.
108. Gawlik B, Druges M, Bianchi M, Bortoli A, Kettrup A, Muntau H. 1997. TUNA FISH (T-30) - A new proficiency testing material for the determination of As and Hg in seafood. Fresenius J Anal Chem 358(3), 441-445.
109. Gerhart EH. 1977. Concentrations of total mercury in several fishes from Delaware Bay, 1975. Pesticides Monitoring Journal 11(3), 132-133.
110. Gerstenberger SL, Martinson A, Kramer JL. 2010. An evaluation of mercury concentrations in three brands of canned tuna. Environ Toxicol Chem 29(2), 237-242.
111. Gilmartin M, Revelante N. 1975. Concentration of Mercury, Copper, Nickel, Silver, Cadmium, and Lead in Northern Adriatic Anchovy, Engraulis encrasicholus, and Sardine, Sardina pilchardus. Fishery Bulletin 73(1), 193-201.
112. Golzadeh, N., Barst, B. D., Basu, N., Baker, J. M., Auger, J C., McKinney, M. A., 2020. Evaluating the concentrations of total mercury, methylmercury, selenium, and selenium:mercury molar ratios in traditional foods of the Bigstone Cree in Alberta, Canada. Chemosphere, 250, 126285.
113. Green NW, Knutzen J. 2003. Organohalogens and metals in marine fish and mussels and some relationships to biological variables at reference localities in Norway. Mar Pollut Bull 46(3), 362-374.
114. Greenfield BK, Jahn A. 2010. Mercury in San Francisco Bay forage fish. Environ Pollut 158(8), 2716-2724.

115. Green-Ruiz C, Ruelas-Inzunza J, Paez-Osuna F. 2005. Mercury in surface sediments and benthic organisms from Guaymas Bay, east coast of the Gulf of California. Environ Geochem Health 27(4), 321-329.
116. Greig RA, Wenzloff DR, Mackenzie CL, Merrill AS, Zdanowicz VS. 1978. Trace metals in sea scallops, Placopecten magellanicus, from eastern United States. Bull Environ Contam Toxicol 19(3), 326-334.
117. Gu, Y.G., Huang, H.H., Lin, Q., 2016. Concentrations and human health implications of heavy metals in wild aquatic organisms captured from the core area of Daya Bay's Fishery Resource Reserve, South China Sea. Environ. Toxicol. Pharmacol. 45, 90-94.
118. Gunsen U. 2004. The residue levels of some toxic metals in different fish species. Indian Veterinary Journal 81(12), 1339-1341.
119. Gutierrez AJ, Lozano G, Gonzalez T, Reguera JI, Hardisson A. 2006. Mercury content in tinned molluscs (mussel, cockle, variegated scallop, and razor shell) normally consumed in Spain, 2005. J Food Prot 69(9), 2237-2240.
120. Haines TA, Komov V, Jagoe CH. 1992. Lake acidity and mercury content of fish in Darwin National Reserve, Russia. Environ Pollut 78(1-3), 107-112.
121. Hajeb P, Jinap S, Fatimah AB, Jamilah B. 2010. Methylmercury in marine fish from Malaysian waters and its relationship to total mercury content. Int J Environ Anal Chem 90(10), 812-820.
122. Hajeb P, Jinap S, Ismail A, Fatimah AB, Jamilah B, Rahim MA. 2009. Assessment of mercury level in commonly consumed marine fishes in Malaysia. Food Control 20(1), 79-84.
123. Hammerschmidt CR, Fitzgerald WF. 2006. Bioaccumulation and trophic transfer of methylmercury in Long Island Sound. Arch Environ Contam Toxicol 51(3), 416-424.
124. Hansson SV, Desforges JP, van Beest FM, Bach L, Halden NM, Sonne C, Mosbech A, Sondergaard, J, 2020. Envion Rese, 183, 109194.
125. Harding G, Dalziel J, Vass P. 2005. Prevalence and bioaccumulation of methylmercury in the food web of the Bay of Fundy, Gulf of Maine. In: The Changing Bay of Fundy - Beyond 400 Years Proceedings of the 6th Bay of Fundy Workshop (Percy JA, Evans AJ, Wells PG, Rolston SJ, eds). Cornwalis, Nova Scotia. September 29 - October 2, 2004. Environment Canada, Atlantic Region, 76-77.
126. Hardisson A, Padron AG, de Bonis A, Sierra A. 1999. Determination of mercury in fish by cold vapor atomic absorption spectrometry. Atom Spectrosc 20(5), 191-193.
127. Hauser-Davis, R.A., Pereira, C.F., Pint, F., Torres, J.P.M., Malm, O., Vianna, M., 2020. Mercury contamination in the recently described Brazilian white-tail dogfish Squalus albicaudus (Squalidae, Chondrichthyes). Chemosphere, 250, 126228.
128. Health Canada. 2007. Human Health Risk Assessment of Mercury in Fish and Health Benefits of Fish Consumption. Ottawa, Ontario. Available: http://www.hc-sc.gc.ca/fnan/pubs/mercur/merc_fish_poisson-eng.php [accessed 31 October 2011].
129. Herreros MA, Inigo-Nunez S, Sanchez-Perez E, Encinas T, Gonzalez-Bulnes A. 2008. Contribution of fish consumption to heavy metals exposure in women of childbearing age from a Mediterranean country (Spain). Food Chem Toxicol 46(5), 1591-1595.
130. Horwitz R, Ashley J, Overbeck P, Velinsky D. 2005. Final Report: Routine Monitoring Program for Toxics in Fish. Trenton, NJ. Available: http://www.state.nj.us/dep/dsr/final-reportroutinemonitoing5-05.pdf [accessed 24 October 2007].

131. Hueter RE, Fong WG, Henderson G, French MF, Manire CA. 1995. Methylmercury concentration in shark muscle by species, size and distribution of sharks in Florida coastal waters. Water Air Soil Pollut 80(1-4), 893-899.
132. Ikem A, Egiebor NO. 2005. Assessment of trace elements in canned fishes (mackerel, tuna, salmon, sardines and herrings) marketed in Georgia and Alabama (United States of America). Journal of Food Composition and Analysis 18(8), 771-787.
133. Ikingura, J.R., Akagi, H., 2003. Total mercury and methylmercury levels in fish from hydroelectric reservoirs in Tanzania. The Science of the total environment, 304, 355–368.
134. International Pacific Halibut Commission. 2003. Methylmercury and Heavy Metal Contaminant Levels in Alaskan Halibut. Available: http://www.iphc.washington.edu/halcom/pubs/rara/2003rara/2k308RARA.pdf [accessed 19 July 2004].
135. Jackson TA, Whittle DM, Evans MS, Muir DCG. 2008. Evidence for mass-independent and massdependent fractionation of the stable isotopes of mercury by natural processes in aquatic ecosystems. Appl Geochem 23(3), 547-571.
136. Jackson TA. 1991. Biological and environmental control of mercury accumulation by fish in lakes and reservoirs of Northern Manitoba, Canada. Can J Fish Aquat Sci 48(12), 2449-2470.
137. Japanese Ministry of Health. 2003. Results of Mercury/Methylmercury in Fishes (Provisional Translation). Available: http://www.mhlw.go.jp/english/wp/other/councils/mercury/index.html [accessed 28 January 2011].
138. Jardine LB, Burt MDB, Arp PA, Diamond AW. 2009. Mercury comparisons between farmed and wild Atlantic salmon (Salmo salar L.) and Atlantic cod (Gadus morhua L.). Aquac Res 40(10), 1148-1159.
139. Jasmine GI, Rajagopalsamy CBT, Jeyachandran P. 1989. Total mercury content of Indian Squid Loligo duvauceli orbigny from Tuticorin waters, south east coast of India. Indian J Mar Sci 18(3), 219-220.
140. Jewett SC, Naidu AS. 2000. Assessment of heavy metals in red king crabs following offshore placer gold mining. Mar Pollut Bull 40(6), 478-490.
141. Jiang, H.F., Qin, D.L., Chen, Z.X., Tang, S.H., Bai, S.Y., Mou, Z.B., 2016. Heavy metal levels in fish from Heilongjiang River and potential health risk assessment. Bull. Environ. Contam. Toxicol. 97, 536-542.
142. Jokai Z, Abranko L, Fodor P. 2005. SPME-GC-pyrolysis-AFS determination of methylmercury in marine fish products by alkaline sample preparation and aqueous phase phenylation derivatization. J Agric Food Chem 53(14), 5499-5505.
143. Julshamn K, Brenna J. 2002. Determination of mercury in seafood by flow injection-cold vapor atomic absorption spectrometry after microwave digestion: NMKL Interlaboratory Study. J AOAC Int 85(3), 626-631.
144. Julshamn K, Grosvik BE, Nedreaas K, Maage A. 2006. Mercury concentration in fillets of Greenland halibut (Reinhardtius hippoglossoides) caught in the Barents Sea in January 2006. Sci Total Environ 372(1), 345-349.
145. Julshamn K, Lundebye AK, Heggstad K, Berntssen MHG, Boe B. 2004. Norwegian monitoring programme on the inorganic and organic contaminants in fish caught in the Barents Sea, Norwegian Sea and North Sea, 1994-2001. Food Addit Contam 21(4), 365-376.

146. Julshamn K, Måge A, Tyssebotn IMB, Sæthre LJ. 2011. Concentrations of Mercury and Other Toxic Elements in Orange Roughy, Hoplostethus atlanticus, from the Mid-Atlantic Ridge. Bull Environ Contam Toxicol 87, 70-73.
147. Juresa D, Blanusa M. 2003. Mercury, arsenic, lead and cadmium in fish and shellfish from the Adriatic Sea. Food Addit Contam 20(3), 241-246.
148. Kai N, Ueda T, Takeda Y, Kataoka A. 1987. Accumulation of mercury and selenium in blue marlin. Nippon Suisan Gakkaishi 53(9), 1697-1697.
149. Kamps LR, Carr R, Miller H. 1972. Total mercury - monomethylmercury content of several species of fish. Bull Environ Contam Toxicol 8(5), 273-279.
150. Kaneko JJ, Ralston NVC. 2007. Selenium and mercury in pelagic fish in the central north pacific near Hawaii. Biol Trace Elem Res 119, 242-254.
151. Kannan K, Smith RG, Lee RF, Windom HL, Heitmuller PT, Macauley JM, et al. 1998. Distribution of total mercury and methyl mercury in water, sediment, and fish from south Florida estuaries. Arch Environ Contam Toxicol 34(2), 109-118.
152. Karouna-Renier NK, Snyder RA, Allison JG, Wagner MG, Rao KR. 2007. Accumulation of organic and inorganic contaminants in shellfish collected in estuarine waters near Pensacola, Florida: Contamination profiles and risks to human consumers. Environ Pollut 145(2), 474-488.
153. Kawaguchi T, Porter D, Bushek D, Jones B. 1999. Mercury in the American oyster Crassostrea virginica in South Carolina, USA, and public health concerns. Mar Pollut Bull 38(4), 324-327.
154. Kehrig HDA, Costa M, Moreira I, Malm O. 2001. Methylmercury and total mercury in estuarine organisms from Rio de Janeiro, Brazil. Environ Sci Pollut Res 8(4), 275-279.
155. Kelso JRM, Frank R. 1974. Organochlorine residues, mercury, copper and cadmium in yellow perch, white bass and smallmouth bass, Long Point Bay, Lake Erie. Trans Am Fish Soc 103(3), 577-581.
156. Khansari FE, Ghazi-Khansari M, Abdollahi M. 2005. Heavy metals content of canned tuna fish. Food Chem 93(2), 293-296.
157. Kidd KA, Hesslein RH, Fudge RJP, Hallard KA. 1995. The influence of trophic level as measured by delta N-15 on mercury concentrations in freshwater organisms. Water Air Soil Pollut 80(1-4), 1011-1015.
158. Kidd, K. a, Stern, G., Lemoalle, J., 2004. Mercury and other contaminants in fish from Lake Chad, Africa. Bulletin of environmental contamination and toxicology, 73, 249–256.
159. Kidd, K.a., Bootsma, H.a., Hesslein, R.H., Lyle Lockhart, W., Hecky, R.E., 2003. Mercury Concentrations in the Food Web of Lake Malawi, East Africa. Journal of Great Lakes Research, 29, 258–266.
160. Knight HT, Olson LJ. 1974. Mercury distribution in american smelt from Lake Michigan. Am Midl Nat 91(2), 451-452.
161. Knobeloch LM, Ziarnik M, Anderson HA, Dodson VN. 1995. Imported sea bass as a source of mercury exposure - a Wisconsin case study. Environ Health Perspect 103(6), 604-606.
162. Knowles TG, Farrington D, Kestin SC. 2003. Mercury in UK imported fish and shellfish and UK-farmed fish and their products. Food Addit Contam 20(9), 813-818.
163. Kojadinovic J, Potier M, Le Corre M, Cosson RP, Bustamante P. 2006. Mercury content in commercial pelagic fish and its risk assessment in the Western Indian Ocean. Sci Total Environ 366(2-3), 688-700.
164. Kojadinovic J, Potier M, Le Corre M, Cosson RP, Bustamante P. 2007. Bioaccumulation of trace elements in pelagic fish from the Western Indian Ocean. Environ Pollut 146(2), 548-566.

165. Koli AK, Williams WR, McClary EB, Wright EL, Burrell TM. 1977. Mercury Levels in Freshwater Fish of State of South-Carolina. Bull Environ Contam Toxicol 17(1), 82-89.

166. Kraepiel AML, Keller K, Chin HB, Malcolm EG, Morel FMM. 2003. Sources and variations of mercury in tuna. Environ Sci Technol 37(24), 5551-5558.

167. Kumar M, Aalbersberg B, Mosley L. 2004. IAS Technical Report Number: 2004/03, Mercury Levels in Fijian Seafoods and Potential Health Implications, Report for World Health Organization.

168. Kutter VT, Mirlean N, Baisch PRM, Kutter MT, Silva E. 2009. Mercury in freshwater, estuarine, and marine fishes from Southern Brazil and its ecological implication. Environ Monit Assess 159(1-4), 35-42.

169. Kwaansa-Ansah, E.E., Agorku, S.E., Nriagu, J.O., 2011. Levels of total mercury in different fish species and sediments from the Upper Volta Basin at Yeji in Ghana. Bulletin of environmental contamination and toxicology, 86, 406–409.

170. Kwoczek M, Szefer P, Hac E. 2006. Essential and toxic elements in seafood available in Poland from different geographical regions. J Agric Food Chem 54(8), 3015-3024.

171. La Colla, N.S., Botte, S.E., Marcovecchio, J.E., 2018. Mercury cycling and bioaccumulation in a changing coastal system: From water to aquatic organisms. Mar. Pollut. Bull. 140, 40-50.

172. Laperdina TG, Askarova OB, Papina TS, Eirikh SS, Sorokovikova LM. 1997. Methodological features of the determination of mercury in fish samples (Using fish from the Kureiskoe Reservoir as an example). J Anal Chem 52(6), 584-589.

173. Leblanc JC,Guerin T,Noel L,Calamassi-Tran G,Volatier JL,Verger P,2005. Dietary exposure estimates of 18 elements from the 1st French Total Diet Study.Food Additives and Contaminants, 22, 624-641.

174. Legrand M, Arp P, Ritchie C, Chan HM. 2005. Mercury exposure in two coastal communities of the Bay of Fundy, Canada. Environ Res 98(1), 14-21.

175. Levine KE, Levine MA, Weber FX, Henderson JP, Grohse PM. 2005. Mercury in an assortment of processed and unprocessed seafood samples. Bull Environ Contam Toxicol 74(5), 973-979.

176. Lewis MA, Quarles RL, Dantin DD, Moore JC. 2004. Evaluation of a Florida coastal golf complex as a local and watershed source of bioavailable contaminants. Mar Pollut Bull 48(3-4), 254-262.

177. Liang, P., Shao, D.D., Wu, S.C., Shi, J.B., Sun, X.L., Wu, F.Y., Lo, S.C.L., Wang, W.-X., Wong, M.H., 2011. The influence of mariculture on mercury distribution in sediments and fish around Hong Kong and adjacent mainland China waters. Chemosphere 82, 1038-1043.

178. Licata P, Trombetta D, Cristani M, Naccari C, Martino D, Calo M, et al. 2005. Heavy metals in liver and muscle of bluefin tuna (Thunnus thynnus) caught in the straits of Messina (Sicily, Italy). Environ Monit Assess 107(1-3), 239-248.

179. Linko RR, Terho K. 1977. Occurrence of methyl mercury in pike and baltic herring from Turku Archipelago. Environ Pollut 14(3), 227-235.

180. Liu, C.B., Hua, X.B., Liu, H.W., Yu, B., Mao, Y.X., Wang, D.Y., Yin, Y.G., Hu, L.G., Shi, J.B., Jiang, G.B., 2018. Tracing aquatic bioavailable Hg in three different regions of China using fish Hg isotopes. Ecotoxicol. Environ. Saf. 150, 327-334.

181. Liu, J.L., Xu, X.R., Yu, S., Cheng, H.F., Hong, Y.G., Feng, X.B., 2014. Mercury pollution in fish from South China Sea: Levels, species-specific accumulation, and possible sources. 131, 160-164.

182. Locascio JV, Rudershausen PJ. 2000. An Evaluation of Mercury Levels in Spotted Seatrout in Torpon Bay, J.N. "Ding" Darling Wildlife Rufuge, Sanibel, Florida, With Reference to Previous Studies. Biological Sciences 63(4), 256-260.

183. Lockhart WL, Stern GA, Low G, Hendzel M, Boila G, Roach P, et al. 2005. A history of total mercury in edible muscle of fish from lakes in northern Canada. Sci Total Environ 351, 427-463.

184. Lodenius M, Seppanen A, Herranen M, 1983. Accumulation of mercury in fish and man from reservoirs in Northern Finland. Water, Air & Soil pollution. 19, 237-246.

185. Lourenco HM, Anacleto P, Afonso C, Ferraria V, Martins MF, Carvalho ML, et al. 2009. Elemental composition of cephalopods from Portuguese continental waters. Food Chem 113(4):1146-1153.

186. Lowenstein J, Burger J, Jeitner C, Amato G, Kolokotronis S, Gochfeld M. 2010. DNA Barcodes Reveal Species-specific Mercury Levels in Tuna Sushi That Pose a Health Risk to Consumers. Biology Letters 6(5), 692-695.

187. Lower Duwamish Waterway Group. 2005. Lower Duwamish Waterway Cleanup: Fish and Crab Tissue Data Report. Available: http://www.ldwg.org/assets/fish_crab_tissue/appendix_adata_tables_final.pdf [accessed 2 December 2010].

188. Luckhurst BE, Prince ED, Llopiz JK, Snodgrass D, Brothers EB. 2006. Evidence of blue marlin (Makaira nigricans) spawning in Bermuda waters and elevated mercury levels in large specimens. Bull Mar Sci 79(3), 691-704.

189. Machiwa, J., 2005. Metals in Nile Perch (Lates niloticus) and Suspended Particulate Matter From Lake Victoria, Tanzania. Journal of Environmental Science and Health, Part A: Toxic/Hazardous Substances & Environmental Engineering, 40, 1809–1822.

190. Madany IM, Wahab AAA, AlAlawi Z. 1996. Trace metals concentrations in marine organisms from the coastal areas of Bahrain, Arabian Gulf. Water Air Soil Pollut 91(3-4), 233-248.

191. Madenjian C, O'Connor D. 2008. Trophic Transfer Efficiency of Mercury to Lake Whitefish Coregonus clupeaformis from its Prey. Bull Environ Contam Toxicol 81(6), 566-570.

192. Magalhaes MC, Costa V, Menezes GM, Pinho MR, Santos RS, Monteiro LR. 2007. Intra- and inter-specific variability in total and methylmercury bioaccumulation by eight marine fish species from the Azores. Mar Pollut Bull 54(10):1654-1662.

193. Marcovecchio JE, Moreno VJ, Perez A. 1986. Bio-magnification of total mercury in Bahia Blanca Estuary shark. Mar Pollut Bull 17(6):276-278.

194. Marcovecchio JE, Moreno VJ, Perez A. 1991. Metal accumulation in tissues of sharks from the Bahia Blanca Estuary, Argentina. Mar Environ Res 31(4):263-274.

195. Marsico ET, Machado MES, Knoff M, Clemente SCS. 2007. Total mercury in sharks along the southern Brazilian Coast. Arq Bras Med Vet Zootec 59(6):1593-1596.

196. Mason RP, Heyes D, Sveinsdottir A. 2006. Methylmercury concentrations in fish from tidal waters of the Chesapeake Bay. Arch Environ Contam Toxicol 51(3):425-437.

197. McArthur T, Butler ECV, Jackson GD. 2003. Mercury in the marine food chain in the Southern Ocean at Macquarie Island: an analysis of a top predator, Patagonian toothfish (Dissostichus eleginoides) and a mid-trophic species, the warty squid (Moroteuthis ingens). Polar Biol 27(1):1-5.

198. McKelvey W, Chang M, Arnason J, Jeffery N, Kricheff J, Kass D. 2010. Mercury and polychlorinated biphenyls in Asian market fish: A response to results from mercury biomonitoring in New York City. Environ Res 110(7):650-657.
199. Meador JP, Ernest DW, Kagley AN. 2005. A comparison of the non-essential elements cadmium, mercury, and lead found in fish and sediment from Alaska and California. Sci Total Environ 339(1-3):189-205.
200. Menasveta P, Siriyong R. 1977. Mercury content of severeal predacious fish in Andaman Sea. Mar Pollut Bull 8(9):200-204.
201. Miller GE, Rowland FS, Steinkru FJ, Grant PM, Guinn VP, Kishore R. 1972. Mercury concentration in museum specimens of tuna and swordfish. Science 175(4026):1121-1122.
202. Ministry of Agriculture Fisheries and Food. 1998. Concentrations of Metals and Other Elements in Marine fish and Shellfish. Available: http://archive.food.gov.uk/maff/archive/food/infsheet/1998/no151/151fish.htm [accessed 18 March 2011].
203. Mol JH, Ramlal JS, Lietar C, Verloo M. 2001. Mercury contamination in freshwater, estuarine, and marine fishes in relation to small-scale gold mining in Suriname, South America. Environ Res 86(2):183-197.
204. Mol S, Kahraman AE, Ulusoy S, 2019. Potential health risks of heavy metals to the Turkish and greek populations via consumption of spiny dogfish and thornback ray from the sea of marmara. Turkish Journal of Fisheries and Aquatic Sciences. 19, 19-27.
205. Monson B.A., Staples, DF., Bhavsar, S.P., Holsen, T.M., Schrank, C.S., Moses, S.K., McGoldrick, D.J., Backus, S.M., Williams, K.A., 2011. Spatiotemporal trends of mercury in walleye and largemouth bassfrom the Laurentian Great Lakes Region. Ecotoxicology, 20, 1555–156.
206. Monteiro LR, Lopes HD. 1990. Merucy content of swordfish, Xiphias gladius, in relation to length, weight, age, and sex. Mar Pollut Bull 21(6):293-296.
207. Mueller CS, Ramelow GJ, Beck JN. 1989. Mercury in the Calcasieu River Lake Complex, Louisiana. Bull Environ Contam Toxicol 42(1):71-80.
208. Nadal M, Ferre-Huguet N, Marti-Cid R, Schuhmacher M, Domingo JL. 2008. Exposure to metals through the consumption of fish and seafood by the population living near the Ebro River in Catalonia, Spain: Health risks. Hum Ecol Risk Assess 14(4):780-795.
209. Nakagawa R, Yumita Y, Hiromoto M. 1997. Total mercury intake from fish and shellfish by Japanese people. Chemosphere 35(12):2909-2913.
210. Nakao M, Seoka M, Nakatani M, Okada T, Miyashita S, Tsukamasa Y, et al. 2009. Reduction of mercury levels in cultured bluefin tuna, Thunnus orientalis, using feed with relatively low mercury levels. Aquaculture 288(3-4):226-232.
211. Nakao M, Seoka M, Tsukamasa Y, Kawasaki K, Ando M. 2007. Possibility for decreasing of mercury content in bluefin tuna Thunnus orientalis by fish culture. Fisheries Science 73(3):724-731.
212. National Marine Fisheries Service. 1975. Southwest Fisheries Center Administrative Report No. 2H, 1975, Mercury in the Pacific Blue Marlin.
213. Nfon E, Cousins IT, Jarvinen O, Mukherjee AB, Verta M, Broman D. 2009. Trophodynamics of mercury and other trace elements in a pelagic food chain from the Baltic Sea. Sci Total Environ 407(24):6267-6274.
214. NOAA (National Oceanic and Atmospheric Administration). 2008. National Status and Trends Mussel Watch Program. Available: http://nsandt.noaa.gov/ [accessed 9 September 2008].

215. Oh KS, Suh J, Park S, Paek OA, Yoon HJ, Kim HY, et al. 2008. Mercury and methylmercury levels in marine fish species from Korean retail markets. Food Sci Biotechnol 17(4):819-823.
216. Onsanit, M. Chen, C. Ke, W.-X. WangMercury and stable isotope signatures in caged marine fish and fish feeds. J. Hazard Mater, 203–204 (2012), pp. 13-21
217. Oppong, S.O.B., Voegborlo, R.B., Agorku, S.E., Adimado, A.A., 2010. Total mercury in fish, sediments and soil from the River Pra Basin, southwestern Ghana. Bulletin of environmental contamination and toxicology, 85, 324–329.
218. Ouédraogo, O., Amyot, M., 2013. Mercury, arsenic and selenium concentrations in water and fish from sub-Saharan semi-arid freshwater reservoirs (Burkina Faso). The Science of the total environment, 444, 243–254.
219. Ozden O. 2010. Seasonal differences in the trace metal and macrominerals in shrimp (Parapenaus longirostris) from Marmara Sea. Environ Monit Assess 162(1-4):191-199.
220. Padula DJ, Daughtry BJ, Nowak BF. 2008. Dioxins, PCBs, metals, metalloids, pesticides and antimicrobial residues in wild and farmed Australian southern bluefin tuna (Thunnus maccoyii). Chemosphere 72(1), 34-44.
221. Pan, K., Chan, H.D., Tam, Y.K., Wang, W.-X., 2014. Low mercury levels in marine fish from estuarine and coastal environments in southern China. Environ. Pollut. 185, 250-257.
222. Panutrakul S, Khamdech S, Kerdthong P, Senanan W, Tangkrock-Olan N, Alcivar-Warren A. 2007. Heavy metals in wild banana prawn (Fenneropenaeus merguiensis de Man, 1888) from Chantaburi and Trat provinces, Thailand. J Shellfish Res 26(4):1193-1202.
223. Papetti P, Rossi G. 2009. Heavy metals in the fishery products of low Lazio and the use of metallothionein as a biomarker of contamination. Environ Monit Assess 159(1-4):589-598.
224. Papu-Zamxaka, V., Mathee, A., Harpham, T., Barnes, B., Röllin, H., Lyons, M., Jordaan, W., Cloete, M., 2010. Elevated mercury exposure in communities living alongside the Inanda Dam, South Africa. Journal of environmental monitoring, 12, 472–477.
225. Park J, Presley BJ. 1997. Trace metals contamination of sediments and organisms from the Swan Lake area of Galveston Bay. Environ Pollut 98(2):209-221.
226. Pastor A, Hernandez F, Peris MA, Beltran J, Sancho JV, Castillo MT. 1994. Levels of heavy metals in some marine organisms from the western Mediterranean area (Spain). Mar Pollut Bull 28(1), 50-53.
227. Paul MC, Toia RF, von Nagy-Felsobuki EI. 2003. A novel method for the determination of mercury and selenium in shark tissue using high-resolution inductively coupled plasma-mass spectrometry. Spectroc Acta Pt B-Atom Spectr 58(9):1687-1697.
228. Payne EJ, Taylor DL. 2010. Effects of Diet Composition and Trophic Structure on Mercury Bioaccumulation in Temperate Flatfishes. Arch Environ Contam Toxicol 58(2):431-443.
229. Penedo de Pinho A, Davee Guimaraes JR, Martins AS, Costa PAS, Olavo G, Valentin J. 2002. Total mercury in muscle tissue of five shark species from Brazilian offshore waters: effects of feeding habit, sex, and length. Environ Res 89(3):250-258.
230. Perello G, Marti-Cid R, Llobet JM, Domingo JL. 2008. Effects of Various Cooking Processes on the Concentrations of Arsenic, Cadmium, Mercury, and Lead in Foods. J Agric Food Chem 56(23):11262-11269.
231. Petersen A, Mortensen GK. 1994. Trace elements in shellfish on the Danish market. Food Addit Contam 11(3):365-373.

232. Pinheiro, M.D.O., Simmons, D.B.D., Villella, M., Tetreaultet, G.R., Muir, D.C.G., McMaster, M.E., Hewitt, L.M., Parrott, J.L., Park, B.J., Brown, S.B., Sherry, J.P., 2020. Brown bullhead at the St. Lawrence River (Cornwall) Area of Concern: health and endocrine status in the context of tissue concentrations of PCBs and mercury. Environ Monit Assess 192, 404.
233. Piraino MN, Taylor DL. 2009. Bioaccumulation and trophic transfer of mercury in striped bass (Morone saxatilis) and tautog (Tautoga onitis) from the Narragansett Bay (Rhode Island, USA). Mar Environ Res 67(3):117-128.
234. Plessi M, Bertelli D, Monzani A. 2001. Mercury and selenium content in selected seafood. Journal of Food Composition and Analysis 14(5):461-467.
235. Polak-Juszczak L. 2009. Temporal trends in the bioaccumulation of trace metals in herring, sprat, and cod from the southern Baltic Sea in the 1994-2003 period. Chemosphere 76(10):1334-1339.
236. Poperechna N, Heumann KG. 2005. Simultaneous multi-species determination of trimethyllead, monomethylmercury and three butyltin compounds by species-specific isotope dilution GC-ICP-MS in biological samples. Anal Bioanal Chem 383(2):153-159.
237. Poste, A.E., Hecky, R.E., Muir, D., 2008. Biomagnification of mercury in a West African crater lake (Lake Bosomtwe, Ghana). Verh. Internat. Verein. Limnol., 30, 647-650.
238. Poste, A.E., Muir, D.C.G., Mbabazi, D., Hecky, R.E. 2012. Food web structure and mercury trophodynamics in two contrasting embayments in northern Lake Victoria. Journal of Great Lakes Research, 38, 699–707.
239. Rahman SA, Wood AK, Sarmani S, Majid AA. 1997. Determination of mercury and organic mercury contents in Malaysian seafood. J Radioanal Nucl Chem 217(1):53-56.
240. Ramlal PS, Bugenyi FWB, Kling GW, Nriagu JO, Rudd JWM, Campbell LM. 2003. Mercury concentrations in water, sediment, and biota from Lake Victoria, East Africa. J Gt Lakes Res 29:283-291.
241. Rasmussen RS, Morrissey MT. 2007. Effects of canning on total mercury, protein, lipid, and moisture content in troll-caught albacore tuna (Thunnus alalunga). Food Chem 101(3):1130-1135.
242. Ray S, Jessop BM, Coffin J, Swetnam DA. 1984. Mercury and Polychlorinated-Biphenyls in Striped Bass (Morone saxatilis) from 2 Nova-Scotia Rivers. Water Air Soil Pollut 21(1-4):15-23.
243. Raymond B, Rossmann R. 2009. Total and methyl mercury accumulation in 1994-1995 Lake Michigan lake trout and forage fish. J Gt Lakes Res 35(3):438-446.
244. Rider S, Adams D. 2000. Mercury Concentrations in Spotted Seatrout from Northwest Florida. Gulf of Mexico Science 2:97-103.
245. Riget F, Moller P, Dietz R, Nielsen TG, Asmund G, Strand J, et al. 2007. Transfer of mercury in the marine food web of West Greenland. J Environ Monit 9(8):877-883.
246. Rivers JB, Pearson JE, Shultz CD. 1972. Total and organic mercury in marine fish. Bull Environ Contam Toxicol 8(5):257-265.
247. Romeo M, Siau Y, Sidoumou Z, Gnassia-Barelli M. 1999. Heavy metal distribution in different fish species from the Mauritania coast. Sci Total Environ 232(3):169-175.
248. Ruelas-Inzunza J, Garcia-Rosales SB, Paez-Osuna F. 2004. Distribution of mercury in adult penaeid shrimps from Altata-Ensenada del Pabellon lagoon (SE Gulf of California). Chemosphere 57(11):1657-1661.
249. Saei-Dehkordi S, Fallah A, Nematollahi A, 2010. Arsenic and mercury in commercially valuable fish species from the PersianGulf: Influence of season and habitat. Food chem. Toxicol. 48, 2945-2950.

250. Sahuquillo I, Lagarda MJ, Silvestre MD, Farre R. 2007. Methylmercury determination in fish and seafood products and estimated daily intake for the Spanish population. Food Addit Contam 24(8):869-876.
251. San Francisco Estuary Institute. 2007. California Bay – Delta Authority Fish Mercury Project: Year 2 Annual Report (Sport Fish Sampling and Analysis). Contribution no. 535. Available: http://legacy.sfei.org/rmp/data/rmpfishtissue.htm [accessed 10 April 2008].
252. San Francisco Public Utilities Commission. 2006. Southwest Ocean Outfall Regional Monitoring Program, Eight Year Summary Report, 1997-2004 San Francisco. Available: http://sfwater.org/main.cfm/MC_ID/4/MSC_ID/83 [accessed 27 January 2011].
253. Santerre CR, Bush PB, Xu DH, Lewis GW, Davis JT, Grodner RM, et al. 2001. Metal residues in farm-raised channel catfish, rainbow trout, and red swamp crayfish from the southern US. J Food Sci 66(2):270-273.
254. Santoyo MM, Figueroa JAL, Wrobel K. 2009. Analytical speciation of mercury in fish tissues by reversed phase liquid chromatography-inductively coupled plasma mass spectrometry with Bi3+ as internal standard. Talanta 79(3):706-711.
255. Schetagne R, Doyon JF, Fournier JJ. 2000. Export of mercury downstream from reservoirs. Sci Total Environ 260(1-3):135-145.
256. Scheuhammer AM, Graham JE. 1999. The bioaccumulation of mercury in aquatic organisms from two similar lakes with differing pH. Ecotoxicology 8(1):49-56.
257. Schuler LJ, Howell JP, Heagler MG. 2000. Mercury concentrations in Louisiana and Chinese crayfish. Bull Environ Contam Toxicol 64(1):27-32.
258. Senn DB, Chesney EJ, Blum JD, Bank MS, Maage A, Shine JP. 2010. Stable Isotope (N, C, Hg) Study of Methylmercury Sources and Trophic Transfer in the Northern Gulf of Mexico. Environ Sci Technol 44(5):1630-1637.
259. Shim SM, Dorworth LE, Lasrado JA, Santerre CR. 2004. Mercury and fatty acids in canned tuna, salmon, and mackerel. J Food Sci 69(9):C681-C684.
260. Shim SM, Lasrado JA, Dorworth LE, Santerre CR. 2005. Mercury and Omega-3 fatty acids in retail fish sandwiches. J Food Prot 68(3):633-635.
261. Shomura R, Craig W. 1974. Mercury in Several Species of Billfishes Taken Off Hawaii and Southern California Kailua-Kona, Hawaii:Proceedings of the International Billfish Symposium.
262. Shultz CD, Crear D. 1976. Distribution of total and organic mercury in 7 tissues of Pacific blue marlin, Makaira nigricans. Pacific Science 30(2):101-107.
263. Silva, S., Oliveira Lima, M., 2020. Mercury in fish marketed in the Amazon Triple Frontier and Health Risk Assessment. Chemosphere. 148, 125989.
264. Silva, S.F., et al., 2019. Seasonal variation of mercury in commercial fishes of the Amazon Triple frontier, western Amazon Basin. Ecol. Indicat. 106, 105549.
265. Soegianto A, Moehammadi N, Irawan B, Affandi M, Hamami. 2010. Mercury concentrations in edible species harvested from Gresik coast, Indonesia and its health risk assessment. Cah Biol Mar 51(1):1-8.
266. Soto-Jimenez MF, Amezcua F, Gonzalez-Ledesma R. 2010. Nonessential Metals in Striped Marlin and Indo-Pacific Sailfish in the Southeast Gulf of California, Mexico: Concentration and Assessment of Human Health Risk. Arch Environ Contam Toxicol 58(3):810-818.

267. Stamatis N, Kamidis N, Pigada P, Stergiou D, Kallianiotis A, 2019. Bioaccumulation Levels and Potential Health Risks of Mercury, Cadmium, and Lead in Albacore (Thunnus alalunga, Bonnaterre, 1788) from The Aegean Sea, Greece. Int J Env Res Pub He, 16, 821.

268. State of Alaska Department of Environmental Conservation. 2009. Total Mercury Concentrations in Alaskan Fishes. Available: http://dec.alaska.gov/eh/docs/vet/metals%2009-11-19.pdf [accessed 26 January 2011].

269. State of Delaware. 2010. Department of Natural Resources and Environmental Control Open Files. Available: http://www.dnrec.state.de.us/fw/advisory.htm [accessed 29 November 2010].

270. State of Louisiana. 2011. Department of Environmental Quality Mercury Fish Tissue Data 2005-2010 (Data file). Available: http://www.deq.louisiana.gov/portal/default.aspx?tabid=1633 [accessed 4 February 2011].

271. State of Maryland. 2007. Toxics Data Sets. Available: http://archive.chesapeakebay.net/data/historicaldb/toxicsmain.htm [accessed 24 October 2007].

272. State of Michigan. 2011. Fish Contaminant Monitoring Program Online Database. Available: http://www.deq.state.mi.us/fcmp/ [accessed 25 February 2011].

273. State of New Jersey. 2004. Routine Monitoring For Toxics in Fish Program Available: http://www.state.nj.us/dep/dsr/task1.pdf (Task I- Appendix I. Draft Summary of Chemical Contaminant Concentrations) and http://www.state.nj.us/dep/dsr/task2.pdf (Task 2) [accessed 29 November 2011].

274. State of New Jersey. 2008. Routine Monitoring Program for Toxics in Fish: Year 3 Raritan River Region. Available: http://www.state.nj.us/dep/dsr/fishmonitoring-year3.pdf [accessed 29 November 2011].

275. State of North Carolina. 2011. Statewide Fish Tissue Metals Results Available: http://portal.ncdenr.org/web/wq/ess/bau/fish-tissue-data, for 1990-2010 [accessed 26 January 2011].

276. State of Virginia. 2009. Department of Environmental Quality Fish Tissue Results Summary. Available: http://www.deq.state.va.us/fishtissue/fishtissue.html [accessed 21 July 2009].

277. Storelli MM, Barone G, Piscitelli G, Marcotrigiano GO. 2007. Mercury in fish: Concentration vs. fish size and estimates of mercury intake. Food Addit Contam 24(12):1353-1357.

278. Storelli MM, Garofalo R, Giungato D, Giacominelli-Stuffler R. 2010. Intake of essential and non-essential elements from consumption of octopus, cuttlefish and squid. Food Addit Contam Part B-Surveill 3(1):14-18.

279. Storelli MM, Giacominelli-Stuffler R, Storelli A, D'Addabbo R, Palermo C, Marcotrigiano GO. 2003. Survey of total mercury and methylmercury levels in edible fish from the Adriatic Sea. Food Addit Contam 20(12):1114-1119.

280. Storelli MM, Giacominelli-Stuffler R, Storelli A, Marcotrigiano GO. 2005. Accumulation of mercury, cadmium, lead and arsenic in swordfish and bluefin tuna from the Mediterranean Sea: A comparative study. Mar Pollut Bull 50(9):1004-1007.

281. Storelli MM, Giacominelli-Stuffler R, Storelli A, Marcotrigiano GO. 2006. Cadmium and mercury in cephalopod molluscs: Estimated weekly intake. Food Addit Contam 23(1):25-30.

282. Storelli MM, Marcotrigiano GO. 2001. Total mercury levels in muscle tissue of swordfish (Xiphias gladius) and bluefin tuna (Thunnus thynnus) from the Mediterranean Sea (Italy). J Food Prot 64(7):1058-1061.

283. Storelli MM, Stuffler RG, Marcotrigiano GO. 1998. Total mercury in muscle of benthic and pelagic fish from the South Adriatic Sea (Italy). Food Addit Contam 15(8):876-883.

284. Storelli MM, Stuffler RG, Marcotrigiano GO. 2002. Total and methylmercury residues in tuna-fish from the Mediterranean sea. Food Addit Contam 19(8):715-720.
285. Strom DG, Graves GA. 2001. A comparison of mercury in estuarine fish between Florida Bay and the Indian River Lagoon, Florida, USA. Estuaries 24(4):597-609.
286. Suk SH, Smith SE, Ramon DA. 2009. Bioaccumulation of mercury in pelagic sharks from the northeast Pacific Ocean. CalCOFI Rep., Vol. 50, 2009. La Jolla, CA. Available: http://calcofi.org/publications/calcofireports/v50/172-177_Suk.pdf [accessed 3 May 2011].
287. Tadiso, T.M., Borgstrøm, R., Rosseland, B.O., 2011. Mercury concentrations are low in commercial fish species of Lake Ziway, Ethiopia, but stable isotope data indicated biomagnification. Ecotoxicology and environmental safety, 74, 953–959.
288. Tariq J, Jaffar M, Ashraf M, 1991. Levels of selected heavy metals in commercial fish from five freshwater lakes, Pakistan, Toxicological & Environmental Chemistry, 33, 1-2, 133-140,
289. Taylor DL, Kutil NJ, Malek AJ, Collie JS. 2014. Mercury bioac-cumulation in cartilaginous fishes from Southern New Eng-land coastal waters: contamination from a trophic ecology and human health perspective. Mar Environ Res 99(4), 20–33.
290. Taylor, H., Appleton, J.D., Lister, R., Smith, B., Chitamweba, D., Mkumbo, O., Machiwa, J. F., Tesha, a L., Beinhoff, C., 2005. Environmental assessment of mercury contamination from the Rwamagasa artisanal gold mining centre, Geita District, Tanzania. The Science of the total environment, 343, 111–133.
291. Teeny FM, Hall AS, Gauglitz EJ. 1974. Reduction of mercury in sablefish (Anoplopoma fimbria) and use of treated flesh in smoked products. Marine Fisheries Review 36(5):15-17.
292. Torres-Escribano S, Velez D, Montoro R. 2010. Mercury and methylmercury bioaccessibility in swordfish. Food Addit Contam Part A-Chem 27(3):327-337.
293. Tuzen, M., Karaman, I., Citak, D., Soylak, M., 2009. Mercury(II) and methyl mercury determinations in water and fish samples by using solid phase extraction and cold vapour atomic absorption spectrometry combination. Food and Chemical Toxicology, 47, 1648–1652.
294. Tyrell L, McHugh B, Glynn D, Twomey M, Joyce E, Costello J, McGovern E, 2005. Trace Metal Concentrations in Various Fish Species Landed at Selected Irish Ports, 2003. Abbotstown, Dublin:Marine Environment and Health Series.
295. USEPA (U.S. Environmental Protection Agency) Region 9 and NOAA (National Oceanic and Atmospheric Administration. 2007. 2002-2004 Southern California Coastal Marine Fish Contaminants Survey. Available: http://earth1.epa.gov/region09/features/pvshelf/montrose_report.pdf [accessed 14 August 2007].
296. USEPA (U.S. Environmental Protection Agency). 2004. Environmental Monitoring and Assessment Program (EMAP). Available: http://www.epa.gov/emap/ (EPA West) [accessed 26 April 2004].
297. USEPA (U.S. Environmental Protection Agency). 2005. Proceedings of the 2005 National Forum on Contaminants in Fish, Analysis of Chemical Contaminant Levels in Store-Bought Fish from Washington State. Available: http://water.epa.gov/scitech/swguidance/fishshellfish/techguidance/upload/2008_11_18_fish_forum_2005_proceedings2005.pdf [accessed 3 November 2011].
298. USEPA (U.S. Environmental Protection Agency). 2006a. Mid-Atlantic Integrated Assessment (MAIA). Available: http://www.epa.gov/emap/maia/html/data/estuary/9798/ [accessed 22 February 2006].

299. USEPA (U.S. Environmental Protection Agency). 2006b. Regional Environmental Monitoring and Assessment Program (REMAP). Available: http://www.epa.gov/emap/remap/html/data.html (Texas, 1993-1994) [accessed 21 June 2006].
300. USEPA (U.S. Environmental Protection Agency). 2007. Environmental Monitoring and Assessment Program (EMAP). Available: http://www.epa.gov/emap/ (Carolinian Province 1994-1997) [accessed 24 October 2007].
301. USEPA (U.S. Environmental Protection Agency). 2008. National Coastal Assessment. Available: http://www.epa.gov/emap/nca/html/data/index.html [accessed 3 October 2008].
302. USEPA (U.S. Environmental Protection Agency). 2011a. National Listing of Fish Advisories. Available: http://map1.epa.gov [accessed 14 September 2011].
303. USEPA (U.S. Environmental Protection Agency). 2011b. Environmental Monitoring and Assessment Program (EMAP). Available: http://www.epa.gov/emap/ (Virginian Province 1991-1993) [accessed 3 February 2011].
304. USEPA (U.S. Environmental Protection Agency). 2011c. Environmental Monitoring and Assessment Program (EMAP). Available: http://www.epa.gov/emap/ (Louisianian Province 1991-1994) [accessed 3 February 2011].
305. USFDA (U.S. Food and Drug Administration). 2008. Total Diet Study 1991-2005. Available: http://www.fda.gov/downloads/Food/FoodSafety/FoodContaminantsAdulteration/TotalDietStudy/UCM243059.pdf [accessed 9 September 2008].
306. USFDA (U.S. Food and Drug Administration). 2014. Mercury Levels in Commercial Fish and Shellfish (1990-2012). Available: https://www.fda.gov/food/metals-and-your-food/mercury-levels-commercial-fish-and-shellfish-1990-2012.
307. Usydus Z, Szlinder-Richert J, Polak-Juszczak L, Komar K, Adamczyk M, Malesa-Ciecwierz M, et al. 2009. Fish products available in Polish market - Assessment of the nutritive value and human exposure to dioxins and other contaminants. Chemosphere 74(11):1420-1428.
308. Vandenbroek WLF. 1981. Concentration and distribution of mercury in flesh of orange roughy (Hoplostethus atlanticus). N Z J Mar Freshw Res 15(3):255-260.
309. Vedrina-Dragojevic I, Dragojevic D, Bujan M. 2002. Total mercury content in fish und molluscs from Adriatic Sea. Dtsch Lebensm-Rundsch 98(1):10-13. 20
310. Vega-Sanchez, B., Ortega-Garcia, S., Ruelas-Inzunza, J., Frias-Espericueta, M., Escobar-Sanchez, O., Guzman-Rendon, J., 2017. Mercury in the Blue Marlin (Makaira nigricans) from the Southern Gulf of California: Tissue Distribution and Inter-Annual Variation (2005-2012). Bull Environ Contam Toxicol 98, 156-161.
311. Viana F, Huertas R, Danulat E. 2005. Heavy metal levels in fish from coastal waters of Uruguay. Arch Environ Contam Toxicol 48(4):530-537.
312. Voegborlo RB, El-Methnani AM, Abedin MZ. 1999. Mercury, cadmium and lead content of canned tuna fish. Food Chem 67(4):341-345.
313. Voegborlo RB, Matsuyama A, Akagi H, Adimado AA, Ephraim JH. 2006. Total mercury and methylmercury accumulation in the muscle tissue of frigate (Auxis thazard thazard) and yellow fin (Thunnus albacares) tuna from the Gulf of Guinea, Ghana. Bull Environ Contam Toxicol 76(5):840-847.

314. Voegborlo, R.B., Akagi, H., 2007. Determination of mercury in fish by cold vapour atomic absorption spectrometry using an automatic mercury analyzer. Food Chem 100, 853-858.
315. Voigt H-R, 2000. Heavy metal organochlorine levels in coastal fishes from the Vaike Vain strait, western Estonia, in high summers og 1993-94. Proc. Estonian Acad. Sci, Bio. Ecol, 49, 4, 335-343.
316. Wang YW, Liang LN, Shi JB, Jiang GB. 2005. Chemometrics methods for the investigation of methylmercury and total mercury contamination in mollusks samples collected from coastal sites along the Chinese Bohai Sea. Environ Pollut 135(3):457-467.
317. Wang, H.S., Xu, W.F., Chen, Z.J., Cheng, Z., Ge, L.C., Man, Y.B., Giesy, J.P., Du, J., Wong, C.K.C., Wong, M.H., 2013a. In vitro estimation of exposure of Hong Kong residents to mercury and methylmercury via consumption of market fishes. J. Hazard Mater. 248, 387-393.
318. Wang, S.J., Dong, D.M, Li, P., Hua, X.Y., Zheng, N., Sun, S.Y., Hou, S.G., An, Q.R., Li, P.Y., Li, Y.Y., Song, X., Li, X.Q., 2020. Mercury concentration and fatty acid composition in muscle tissue of marine fish species harvested from Liaodong Gulf: An intelligence quotient and coronary heart disease risk assessment. Sci. Total Environ. 726, 138586.
319. Watanabe T, Hayashi T, Matsuda R, Akiyama H, Teshima R. 2017. Shokuhin Eiseigaku Zasshi. 58(2):80-85. doi:10.3358/shokueishi.58.80
320. Watling RJ, McClurg TP, Stanton RC. 1981. Relation between mercury concentration and size in the mako shark. Bull Environ Contam Toxicol 26(3):352-358.
321. Whyte ALH, Hook GR, Greening GE, Gibbs-Smith E, Gardner JPA. 2009. Human dietary exposure to heavy metals via the consumption of greenshell mussels (Perna canaliculus Gmelin 1791) from the Bay of Islands, northern New Zealand. Sci Total Environ 407(14):4348-4355.
322. Wren CD, Scheider WA, Wales DL, Muncaster BW, Gray IM. 1991. Relation between Mercury Concentrations in Walleye (Stizostedion vitreum vitreum) and Northern Pike (Esox lucius) in Ontario Lakes and Influence of Environmental-Factors. Can J Fish Aquat Sci 48(1):132-139.
323. Xu, X.Y., Wang, W.-X., 2017. Mercury exposure and source tracking in distinct marine-caged fish farm in southern China. Environ. Pollut. 220, 1138-1146.
324. Yamashita Y, Omura Y, Okazaki E. 2005. Total mercury and methylmercury levels in commercially important fishes in Japan. Fisheries Science 71(5):1029-1035.
325. Yamashita Y, Omura Y, Okazaki E. 2006. Distinct regional profiles of trace element content in muscle of Japanese eel Anguilla japonica from Japan, Taiwan, and China. Fisheries Science 72(5):1109-1113.
326. Yang, R.Q., Jing, C.Y., Zhang, Q.H., Jiang, G.B., 2013. Identifying semi-volatile contaminants in fish from niyang river, Tibetan plateau. Environ. Earth Sci. 68, 1065-1072.
327. Yoshino, K., Mori, K., Kanaya, G., Kojima, S, Henmi, Y., Matsuyama, A., Yamamoto, M., 2020. Food sources are more important than biomagnification on mercury bioaccumulation in marine fishes, Environmental Pollution, 296, 113982.
328. Zauke GP, Savinov VM, Ritterhoff J, Savinova T. 1999. Heavy metals in fish from the Barents Sea in (summer 1994). Sci Total Environ 227(2-3):161-173.
329. Zhang XM, Naidu AS, Kelley JJ, Jewett SC, Dasher D, Duffy LK. 2001. Baseline concentrations of total mercury and methylmercury in salmon returning via the Bering Sea (1999-2000). Mar Pollut Bull 42(10):993-997.

330. Zhang, W., Zhang, X., Tian, Y.L., Zhu, Y., Tong, Y.D., Li, Y., Wang, X.J., 2018. Risk assessment of total mercury and methylmercury in aquatic products from offshore farms in China. J. Hazard Mater. 354, 198-205.
331. Zhu, L.L., Yan, B.X., Wang, L.X., Pan, X.F., 2012. Mercury concentration in the muscle of seven fish species from Chagan Lake, Northeast China. Environ. Monit. Assess. 184, 1299-1310.

Rice

1. Al-Saleh, I. & Abduljabbar, M. Heavy metals (lead, cadmium, methylmercury, arsenic) in commonly imported rice grains (Oryza sativa) sold in Saudi Arabia and their potential health risk. Int. J. Hyg. Environ. Health. 220, 1168-1178 (2017).
2. Al-Saleh, I. & Shinwari, N. Report on the levels of cadmium, lead, and mercury in imported rice grain samples. Biol. Trace Elem. Res. 83, 91-96 (2001).
3. Appleton, J., Weeks, J., Calvez, J. & Beinhoff, C. Impacts of mercury contaminated mining waste on soil quality, crops, bivalves, and fish in the Naboc River area, Mindanao, Philippines. Sci. Total Environ. 354, 198-211 (2006).
4. Batista, B. L., Nacano, L. R., de Freitas, R., de Oliveira-Souza, V. C. & Barbosa, F. Determination of essential (Ca, Fe, I, K, Mo) and toxic elements (Hg, Pb) in Brazilian rice grains and estimation of reference daily intake. Pol. J. food Nutr. Sci. 3, 129 (2012).
5. Bennett, J. P., Chiriboga, E., Coleman, J. & Waller, D. M. Heavy metals in wild rice from northern Wisconsin. Sci. Total Environ. 246, 261-269 (2000).
6. Brombach, C.-C. et al. Methylmercury varies more than one order of magnitude in commercial European rice. Food Chem. 214, 360-365 (2017).
7. Cao, H., Chen, J., Zhang, J., Zhang, H., Qiao, L., Men, Y. Heavy metals in rice and garden vegetables and their potential health risks to inhabitants in the vicinity of an industrial zone in Jiangsu, China. J. Environ. Sci. 22, 1792-1799 (2010).
8. Cheng, J., Zhao, W., Wang, Q., Liu, X., Wang, W., 2013a. Accumulation of mercury, selenium and PCBs in domestic duck brain, liver and egg from a contaminated area with an investigation of their redox responses. Environmental Toxicology and Pharmacology 35, 388-394.
9. Cheng, Z., Wang, H.S., Du, J., Sthiannopkao, S., Xing, G.H., Kim, K.W., Yasin, M.S., Hashim, J.H., Wong, M.H., 2013b. Dietary exposure and risk assessment of mercury via total diet study in Cambodia. Chemosphere 92, 143-149.
10. Cui, W., Liu, G., Bezerra, M., Lagos, D.A., Li, Y., Cai, Y., 2017. Occurrence of Methylmercury in Rice-Based Infant Cereals and Estimation of Daily Dietary Intake of Methylmercury for Infants. J Agric Food Chem 65, 9569-9578.
11. Da Silva, M. J., Paim, A. P. S., Pimentel, M. F., Cervera, M. L. & De la Guardia, M. Determination of mercury in rice by cold vapor atomic fluorescence spectrometry after microwave-assisted digestion. Anal. Chim. Acta 667, 43-48 (2010).
12. Da, S.D., Portugal, L.A., Serra, A.M., Ferreira, S.L., Cerdà, V., 2013. Determination of mercury in rice by MSFIA and cold vapour atomic fluorescence spectrometry. Food Chemistry 137, 159-163.
13. Fang, Y., Sun, X., Yang, W., Ma, N., Xin, Z., Fu, J., Liu, X., Liu, M., Mariga, A.M., Zhu, X., Hu, Q., 2014. Concentrations and health risks of lead, cadmium, arsenic, and mercury in rice and edible mushrooms in China. Food Chemistry 147, 147-151.

14. Feng, X., Li, P., Qiu, G., Wang, S., Li, G., Shang, L., Meng, B., Jiang, H., Bai, W., Li, Z., 2008. Human exposure to methylmercury through rice intake in mercury mining areas, Guizhou province, China. Environmental Science & Technology 42, 326-332.
15. Horvat, M., Nolde, N., Fajon, V., Jereb, V., Logar, M., Lojen, S., Jacimovic, R., Falnoga, I., Liya, Q., Faganeli, J., 2003. Total mercury, methylmercury and selenium in mercury polluted areas in the province Guizhou, China. Science of the Total Environment 304, 231-256.
16. Huang, Z., Pan, X.D., Wu, P.G., Han, J.L., Chen, Q., 2013. Health risk assessment of heavy metals in rice to the population in Zhejiang, China. PLoS One 8, e75007.
17. Jallad, K.N., 2015. Heavy metal exposure from ingesting rice and its related potential hazardous health risks to humans. Environ Sci Pollut Res Int 22, 15449-15458.
18. Krisnayanti, B. D. et al. Assessment of environmental mercury discharge at a four-year-old artisanal gold mining area on Lombok Island, Indonesia. J. Environ. Monit. 14, 2598-2607 (2012).
19. Leblanc, J.-C. et al. Dietary exposure estimates of 18 elements from the 1st French Total Diet Study. Food Addit. Contam. 22, 624-641 (2005).
20. Lee, D., Thomas, B., Roughan, J. & Watters, E. Mercury content of some foodstuffs of vegetable origin. Pestic. Sci. 3, 13-17 (1972).
21. Lee, H.-S. et al. Dietary exposure of the Korean population to arsenic, cadmium, lead and mercury. J. Food Compos. Anal. 19, S31-S37 (2006).
22. Lenka, M.,Panda,K.,Panda,B.,1992.Monitoring and assessment of mercury pollution in the vicinity of achloroalkali plant. IV. Bioconcentration of mercury in in situ aquatic and terrestrial plants at Ganjam, India. Arch. Environ. Contam. Toxicol. 22, 195–202.
23. Li, P., Feng, X., Qiu, G., Shang, L., Wang, S., 2008. Mercury exposure in the population from Wuchuan mercury mining area, Guizhou, China. Science of the Total Environment 395, 72-79.
24. Li, P., Feng, X., Qiu, G., Shang, L., Li, G., 2009. Human hair mercury levels in the Wanshan mercury mining area, Guizhou Province, China. Environmental Geochemistry and Health 31, 683.
25. Li, P., Feng, X., Shang, L., Qiu, G., Meng, B., Zhang, H., Guo, Y., Liang, P., 2011. Human co-exposure to mercury vapor and methylmercury in artisanal mercury mining areas, Guizhou, China. Ecotoxicology and Environmental Safety 74, 473-479.
26. Li, P., Feng, X., Yuan, X., Chan, H., Qiu, G., Sun, G., Zhu, Y., 2012. Rice consumption contributes to low level methylmercury exposure in southern China. Environmental International 49, 18-23.
27. Li, R., Xu, X., Xu, Z., Ao, M., Shang, L., Qiu, G., Tang, S., 2016. Total Mercury and Methylmercury Concentrations and Risk Assessments in Rice Plants Collected from a River Watershed in a Typical Mercury Mining Area of Guizhou. Res of Environ Sci 29, 1829-1839.
28. Liang, P., Feng, X., Zhang, C., Zhang, J., Cao, Y., You, Q., Leung, A.O.W., Wong, M.-H., Wu, S.-C., 2015. Human exposure to mercury in a compact fluorescent lamp manufacturing area: By food (rice and fish) consumption and occupational exposure. Environmental Pollution 198, 126-132.
29. Maramba et al., 2006. Environmental and human exposure assessment monitoring of communities near an abandoned mercury mine in the Philippines: a toxic legacy. J. Environ. Manage. 81, 135–145.
30. Morishita, T., Kishino, K. & Idaka, S. Mercury contamination of solls, rice plants, and human hair in the vicinity of a mercury mine in mie prefecture, Japan. Soil Sci. Plant Nutr. 28, 523-534 (1982).

31. Nakagawa, R., Yumita, Y., 1998. Change and behavior of residual mercury in paddy soils and rice of Japan. Chemosphere 37, 1483–1487.
32. Nardi, E. P. et al. The use of inductively coupled plasma mass spectrometry (ICP-MS) for the determination of toxic and essential elements in different types of food samples. Food Chem. 112, 727-732 (2009).
33. Pataranawat, P., Parkpian, P., Polprasert, C., Delaune, R.D., Jugsujinda, A., 2007. Mercury emission and distribution: potential environmental risks at a small-scale gold mining operation, Phichit Province, Thailand. J. Environ. Sci. Health. Part A Toxic/Hazard. Subst. Environ. Eng. 42, 1081–1093.
34. Qian, Y., Chen, C., Zhang, Q., Li, Y., Chen, Z., Li, M., 2010. Concentrations of cadmium, lead, mercury and arsenic in Chinese market milled rice and associated population health risk. Food Control 21, 1757-1763.
35. Qiu, G., Feng, X., Li, P., Wang, S., Li, G., Shang, L., Fu, X., 2008. Methylmercury accumulation in rice (Oryza sativa L.) grown at abandoned mercury mines in Guizhou, China. Journal of Agricultural and Food Chemistry 56, 2465-2468.
36. Qiu, G., Feng, X., Meng, B., Sommar, J., Gu, C., 2012. Environmental geochemistry of an active Hg mine in Xunyang, Shaanxi Province, China. Applied Geochemistry 27, 2280-2288.
37. Qiu, G., Feng, X., Meng, B., Zhang, C., Gu, C., Du, B., Lin, Y., 2013. Environmental geochemistry of an abandoned mercury mine in Yanwuping, Guizhou Province, China. Environmental Research 125, 124-130.
38. Rothenberg, S.E., Feng, X., Zhou, W., Tu, M., Jin, B., You, J., 2012. Environment and genotype controls on mercury accumulation in rice (Oryza sativa L.) cultivated along a contamination gradient in Guizhou, China. Science of the Total Environment 426, 272-280.
39. Rothenberg, S.E., Mgutshini, N.L., Bizimis, M., Johnson-Beebout, S.E., Ramanantsoanirina, A., 2015. Retrospective study of methylmercury and other metal(loid)s in Madagascar unpolished rice (Oryza sativa L.). Environmental Pollution 196, 125-133.
40. Rothenberg, S.E., Anders, M., Ajami, N.J., Petrosino, J.F., Balogh, E. 2016. Water management impacts rice methylmercury and the soil microbiome. Sci. Total Environ. 572, 608-617.
41. Rothenberg, S. E. et al. Stable mercury isotopes in polished rice (Oryza Sativa L.) and hair from rice consumers. Environ. Sci. Technol. 51, 6480-6488 (2017).
42. Samal, L., & Garg, A. (2012). Status of toxic heavy metals in cereal grains and pulses in Bareilly district of Uttar Pradesh. Indian Veterinary Journal, 88, 25–27.
43. Sarkar, A., Aronson, K.J., Patil, S., Hugar, L.B., VanLoon, G.W., 2012. Emerging health risks associated with modern agriculture practices: a comprehensive study in India. Environ. Res. 115, 37–50.
44. Shi, J., Liang, L., Jiang, G., 2005. Simultaneous Determination of Methylmercury and Ethylmercury in Rice by Capillary Gas Chromatography Coupled On-line with Atomic Fluorescence Spectrometry. J. AOAC Int. 88, 665-669.
45. Silva, L. O. B., da Silva, D. G., Leao, D. J., Matos, G. D. & Ferreira, S. L. C. Slurry sampling for the determination of mercury in rice using cold vapor atomic absorption spectrometry. Food Anal. Meth. 5, 1289-1295 (2012).
46. Srikumar, T.S., 1993. The mineral and trace element composition of vegetables, pulses and cereals of southern India. Food Chem. 46, 163–167.
47. Taylor,H. et al., 2005. Environmental assessment of mercury contamination from the Rwamagasa artisanal gold mining centre, Geita District, Tanzania. Sci. Total Environ. 343, 111–133.
48. Teherani, D. Trace elements analysis in rice. J. Radioanal. Nucl. Chem. 117, 133-143 (1987).

49. Wang, G., Gong, Y., Zhu, Y.X., Miao, A.J., Yang, L.Y., Zhong, H., 2017. Assessing the Risk of Hg Exposure Associated with Rice Consumption in a Typical City (Suzhou) in Eastern China. International Journal of Environmental Research & Public Health 14, 525.
50. Wang, X., Zeng, X., Chuanping, L., Li, F., Xu, X., & Lv, Y. (2016). Heavy metal contaminations in soil-rice system: Source identification in relation to a sulfur-rich coal burning power plant in northern guangdong province, China. Environmental Monitoring and Assessment, 188, 460.
51. Wang, Y., et al., Total mercury and methylmercury in rice: Exposure and health implications in Bangladesh. Environmental Pollution, 2020. 265: p. 114991.
52. Windham-Myers et al.,2014. Mercury cycling in agricultural and managed wetlands, Yolo Bypass, California: seasonal influences of vegetation on mercury methylation, storage, and transport. Sci. Total Environ. 484,308–318.
53. Xu, X., Meng, B., Zhang, C., Feng, X., Gu, C., Guo, J., Bishop, K., Xu, Z., Zhang, S., Qiu, G., 2017. The local impact of a coal-fired power plant on inorganic mercury and methyl-mercury distribution in rice (Oryza sativa L.) Environmental Pollution 223, 11.
54. Xu, X., Lin, Y., Meng, B., Feng, X., Xu, Z., Jiang, Y., Zhong, W., Hu, Y., Qiu, G., 2018. The impact of an abandoned mercury mine on the environment in the Xiushan region, Chongqing, southwestern China. Applied Geochemistry 88, 267-275.
55. Xu, X., Han, J., Abeysinghe, K.S., Atapattu, A.J., De Silva, P.M.C.S., Xu, Z., Long, S., Qiu, G., 2020a. Dietary exposure assessment of total mercury and methylmercury in commercial rice in Sri Lanka. Chemosphere 239, 124749.
56. Xu, X., et al., Methylmercury and inorganic mercury in Chinese commercial rice: Implications for overestimated human exposure and health risk. Environmental Pollution, 2020b. 258: p. 113706.
57. Zarcinas, B. A., Ishak, C. F., McLaughlin, M. J. & Cozens, G. Heavy metals in soils and crops in Southeast Asia. Environ. Geochem. Health 26, 343-357 (2004a).
58. Zarcinas, B. A., Pongsakul, P., McLaughlin, M. J. & Cozens, G. Heavy metals in soils and crops in Southeast Asia 2. Thailand. Environ. Geochem. Health 26, 359-371 (2004b).
59. Zhang, H., Feng, X., Larssen, T., Qiu, G., Vogt, R.D., 2010a. In inland China, rice, rather than fish, is the major pathway for methylmercury exposure. Environmental Health Perspectives 118, 1183-1188.
60. Zhang, H., Feng, X., Larssen, T., Shang, L., Li, P., 2010b. Bioaccumulation of Methylmercury versus Inorganic Mercury in Rice (Oryza sativa L.) Grain. Environmental Science & Technology 44, 4499-4504.
61. Zhang, H., Wang, D., Zhang, J., Shang, X., Zhao, Y., Wu, Y., 2014. Total mercury in milled rice and brown rice from China and health risk evaluation. Food Addit Contam Part B Surveill 7, 141-146.
62. Zhao, G., Zhou, H., Wang, Z., 2010. Concentrations of selected heavy metals in food from four e-waste disassembly localities and daily intake by local residents. J Environ Sci Health A Tox Hazard Subst Environ Eng 45, 824-835.
63. Zhao, H., et al., Mercury contents in rice and potential health risks across China. Environment International, 2019. 126: p. 406-412.
64. Zhao, X., et al., Total mercury and methylmercury in Chinese rice and dietary exposure assessment. Food additives & contaminants Part B, Surveillance communications, 2020. 13(2): p. 148-153.

Human Biomarkers

1. Airaksinen, R., Turunen, A. W., Rantakokko, P., Männistö, S., Vartiainen, T., & Verkasalo, P. K.

(2010). Blood concentration of methylmercury in relation to food consumption. Public Health Nutrition, 14(03), 480–489.doi:10.1017/s1368980010001485

2. Abbas, H., Sakakibara, M., Sera, K., & Arma, L. (2017). Mercury Exposure and Health Problems in Urban Artisanal Gold Mining (UAGM) in Makassar, South Sulawesi, Indonesia. Geosciences, 7(3), 44.doi:10.3390/geosciences7030044
3. Abdelouahab, N., Mergler, D., Takser, L., Vanier, C., St-Jean, M., Baldwin, M., … Chan, H. M. (2008). Gender differences in the effects of organochlorines, mercury, and lead on thyroid hormone levels in lakeside communities of Quebec (Canada). Environmental Research, 107(3), 380–392. doi:10.1016/j.envres.2008.01.006
4. Abdul Samad, N., Md Isa, Z., & Hod, R. (2017). Mercury Hair Concentration among Primary School Children in Malaysia. Children, 4(12), 109.doi:10.3390/children4120109
5. Agusa, T., Kunito, T., Iwata, H., Monirith, I., Tana, T. S., Subramanian, A., & Tanabe, S. (2005). Mercury contamination in human hair and fish from Cambodia: levels, specific accumulation and risk assessment. Environmental Pollution, 134(1), 79–86.doi:10.1016/j.envpol.2004.07.015
6. Akira, Y., Miyuki, M., Masako, Y., & Noriyuki, H. (2004). Current Hair Mercury Levels in Japanese for Estimation of Methylmercury Exposure. JOURNAL OF HEALTH SCIENCE, 50(2), 120–125. doi:10.1248/jhs.50.120
7. Albatanony, M. A., Abdelrasul, G. M., Abusalem, M. A., Aldalatony, M. M., & Allam, H. K. (2013). Occupational exposure to mercury among workers in a fluorescent lamp factory, Quisna Industrial Zone, Egypt.. The international journal of occupational and environmental medicine, 4(3), 149-156.
8. Al-Saleh, I., & Al-Sedairi, A. anoud. (2011). Mercury (Hg) burden in children: The impact of dental amalgam. Science of The Total Environment, 409(16), 3003–3015.doi:10.1016/j.scitotenv.2011.04.047
9. Al-Saleh, I., Abduljabbar, M., Al-Rouqi, R., Eltabache, C., Al-Rajudi, T., Elkhatib, R., & Nester, M. (2015). The extent of mercury (Hg) exposure among Saudi mothers and their respective infants. Environmental Monitoring and Assessment, 187(11).doi:10.1007/s10661-015-4858-y
10. Al-Saleh, I., Coskun, S., Mashhour, A., Shinwari, N., El-Doush, I., Billedo, G., … El Din Mohamed, G. (2008). Exposure to heavy metals (lead, cadmium and mercury) and its effect on the outcome of in-vitro fertilization treatment. International Journal of Hygiene and Environmental Health, 211(5-6), 560–579. doi:10.1016/j.ijheh.2007.09.005
11. Al-Saleh, I., Shinwari, N., Mashhour, A., Mohamed, G. E. D., & Rabah, A. (2011). Heavy metals (lead, cadmium and mercury) in maternal, cord blood and placenta of healthy women. International Journal of Hygiene and Environmental Health, 214(2), 79–101.doi:10.1016/j.ijheh.2010.10.001
12. Alves, M. F. A., Fraiji, N. A., Barbosa, A. C., De Lima, D. S. N., Souza, J. R., Dórea, J. G., & Cordeiro, G. W. O. (2006). Fish consumption, mercury exposure and serum antinuclear antibody in Amazonians. International Journal of Environmental Health Research, 16(4), 255–262.doi:10.1080/09603120600734147
13. Anwar, M., Ando, T., Maaz, A., Ghani, S., Munir, M., Qureshi, I. U., ... & Akiba, S. (2007). Scalp hair mercury concentrations in Pakistan.. Environmental sciences : an international journal of environmental physiology and toxicology, 14(4), 167-175.
14. Ashe, K. (2012). Elevated Mercury Concentrations in Humans of Madre de Dios, Peru. PLoS ONE, 7(3), e33305.doi:10.1371/journal.pone.0033305

15. Baek, H.-J., Kim, E.-K., Lee, S. G., Jeong, S.-H., Sakong, J., Merchant, A. T., … Choi, Y.-H. (2016). Dental amalgam exposure can elevate urinary mercury concentrations in children. International Dental Journal, 66(3), 136–143. doi:10.1111/idj.12214
16. Baeuml, J., Bose-O'Reilly, S., Gothe, R. M., Lettmeier, B., Roider, G., Drasch, G., & Siebert, U. (2011). Human Biomonitoring Data from Mercury Exposed Miners in Six Artisanal Small-Scale Gold Mining Areas in Asia and Africa. Minerals, 1(1), 122–143.doi:10.3390/min1010122
17. Bahia, M. de O., Corvelo, T. C., Mergler, D., Burbano, R. R., Lima, P. D. L., Cardoso, P. C. S., … Amorim, M. I. M. (2004). Environmental biomonitoring using cytogenetic endpoints in a population exposed to mercury in the Brazilian Amazon. Environmental and Molecular Mutagenesis, 44(4), 346–349. doi:10.1002/em.20054
18. Barbieri, F. L., Cournil, A., & Gardon, J. (2009). Mercury exposure in a high fish eating Bolivian Amazonian population with intense small-scale gold-mining activities. International Journal of Environmental Health Research, 19(4), 267–277.doi:10.1080/09603120802559342
19. Barcelos, G. R. M., Souza, M. F. de, Oliveira, A. Á. S. de, Lengert, A. van H., Oliveira, M. T. de, Camargo, R. B. de O. G., … Barbosa, F. (2015). Effects of genetic polymorphisms on antioxidant status and concentrations of the metals in the blood of riverside Amazonian communities co-exposed to Hg and Pb. Environmental Research, 138, 224–232. doi:10.1016/j.envres.2015.02.017
20. Barghi, M., Behrooz, R. D., Esmaili-Sari, A., & Ghasempouri, S. M. (2012). Mercury Exposure Assessment in Iranian Pregnant Women's Hair with Respect to Diet, Amalgam Filling, and Lactation. Biological Trace Element Research, 148(3), 292–301.doi:10.1007/s12011-012-9384-y
21. Basri, Sakakibara, M., & Sera, K. (2017). Current Mercury Exposure from Artisanal and Small-Scale Gold Mining in Bombana, Southeast Sulawesi, Indonesia—Future Significant Health Risks. Toxics, 5(1), 7.doi:10.3390/toxics5010007
22. Bates, C. J., Prentice, A., Birch, M. C., & Delves, H. T. (2007). Dependence of blood indices of selenium and mercury on estimated fish intake in a national survey of British adults. Public Health Nutrition, 10(05).doi:10.1017/s1368980007246683
23. Bellanger, M., Pichery, C., Aerts, D., Berglund, M., Castaño, A., … Grandjean, P. (2013). Economic benefits of methylmercury exposure control in Europe: Monetary value of neurotoxicity prevention. Environmental Health, 12(1).doi:10.1186/1476-069x-12-3
24. Benefice, E., Luna Monrroy, S. J., & Lopez Rodriguez, R. W. (2008). A nutritional dilemma: fish consumption, mercury exposure and growth of children in Amazonian Bolivia. International Journal of Environmental Health Research, 18(6), 415–427.doi:10.1080/09603120802272235
25. Bentzen, R., Castellini, J. M., Gaxiola-Robles, R., Zenteno-Savín, T., Méndez-Rodríguez, L. C., & O'Hara, T. (2014). Relationship between self-reported fish and shellfish consumption, carbon and nitrogen stable isotope values and total mercury concentrations in pregnant women (II) from Baja California Sur, Mexico. Toxicology Reports, 1, 1115–1122.doi:10.1016/j.toxrep.2014.09.014
26. Bevan, R., Jones, K., Cocker, J., Assem, F. L., & Levy, L. S. (2013). Reference ranges for key biomarkers of chemical exposure within the UK population. International Journal of Hygiene and Environmental Health, 216(2), 170–174.doi:10.1016/j.ijheh.2012.03.005
27. Birgisdottir, B. E., Knutsen, H. K., Haugen, M., Gjelstad, I. M., Jenssen, M. T. S., Ellingsen, D. G., … Brantsæter, A. L. (2013). Essential and toxic element concentrations in blood and urine and their

associations with diet: Results from a Norwegian population study including high-consumers of seafood and game. Science of The Total Environment, 463-464, 836–844.doi:10.1016/j.scitotenv.2013.06.078

28. Björnberg, K. A., Vahter, M., Grawé, K. P., & Berglund, M. (2005). Methyl mercury exposure in Swedish women with high fish consumption. Science of The Total Environment, 341(1-3), 45–52. doi:10.1016/j.scitotenv.2004.09.033
29. Bocca, B., Bena, A., Pino, A., D'Aversa, J., Orengia, M., Farina, E., … Alimonti, A. (2016). Human biomonitoring of metals in adults living near a waste-to-energy incinerator in ante-operam phase: Focus on reference values and health-based assessments. Environmental Research, 148, 338–350.doi:10.1016/j.envres.2016.04.013
30. Bonsignore, M., Andolfi, N., Barra, M., Madeddu, A., Tisano, F., Ingallinella, V., … Sprovieri, M. (2016). Assessment of mercury exposure in human populations: A status report from Augusta Bay (southern Italy). Environmental Research, 150, 592–599.doi:10.1016/j.envres.2016.01.016
31. Bose-O'Reilly, S., Drasch, G., Beinhoff, C., Rodrigues-Filho, S., Roider, G., Lettmeier, B., … Siebert, U. (2010). Health assessment of artisanal gold miners in Indonesia. Science of The Total Environment, 408(4), 713–725.doi:10.1016/j.scitotenv.2009.10.070
32. Bose-O'Reilly, S., Drasch, G., Beinhoff, C., Tesha, A., Drasch, K., Roider, G., … Siebert, U. (2010). Health assessment of artisanal gold miners in Tanzania. Science of The Total Environment, 408(4), 796–805.doi:10.1016/j.scitotenv.2009.10.051
33. Bourdineaud, J.-P., Durrieu, G., Sarrazin, S. L. F., da Silva, W. C. R., Mourão, R. H. V., & de Oliveira, R. B. (2015). Mercurial exposure of residents of Santarém and Oriximiná cities (Pará, Brazil) through fish consumption. Environmental Science and Pollution Research, 22(16), 12150–12161. doi:10.1007/s11356-015-4502-y
34. Buck Louis, G. M., Sundaram, R., Schisterman, E. F., Sweeney, A. M., Lynch, C. D., Gore-Langton, R. E., … Barr, D. B. (2012). Heavy metals and couple fecundity, the LIFE Study. Chemosphere, 87(11), 1201–1207.doi:10.1016/j.chemosphere.2012.01.017
35. Cace, I. B., Milardovic, A., Prpic, I., Krajina, R., Petrovic, O., Vukelic, P., … Snoj, J. (2011). Relationship between the prenatal exposure to low-level of mercury and the size of a newborn's cerebellum. Medical Hypotheses, 76(4), 514–516.doi:10.1016/j.mehy.2010.12.005
36. Carneiro, M. F. H., Moresco, M. B., Chagas, G. R., de Oliveira Souza, V. C., Rhoden, C. R., & Barbosa, F. (2011). Assessment of Trace Elements in Scalp Hair of a Young Urban Population in Brazil. Biological Trace Element Research, 143(2), 815–824.doi:10.1007/s12011-010-8947-z
37. Castaño, A., Sánchez-Rodríguez, J. E., Cañas, A., Esteban, M., Navarro, C., Rodríguez-García, A. C., … Jiménez-Guerrero, J. A. (2012). Mercury, lead and cadmium levels in the urine of 170 Spanish adults: A pilot human biomonitoring study. International Journal of Hygiene and Environmental Health, 215(2), 191–195.doi:10.1016/j.ijheh.2011.09.001
38. Castilhos, Z., Rodrigues-Filho, S., Cesar, R., Rodrigues, A. P., Villas-Bôas, R., de Jesus, I., … Santos, E. (2015). Human exposure and risk assessment associated with mercury contamination in artisanal gold mining areas in the Brazilian Amazon. Environmental Science and Pollution Research, 22(15), 11255–11264. doi:10.1007/s11356-015-4340-y
39. Chang, J., Park, J., Shin, S., Yang, H., Moon, J., & Ko, J. (2015). Mercury Exposure in Healthy Korean Weaning-Age Infants: Association with Growth, Feeding and Fish Intake. International Journal of

Environmental Research and Public Health, 12(11), 14669–14689.doi:10.3390/ijerph121114669

40. Chang, J.-W., Pai, M.-C., Chen, H.-L., Guo, H.-R., Su, H.-J., & Lee, C.-C. (2008). Cognitive function and blood methylmercury in adults living near a deserted chloralkali factory. Environmental Research, 108(3), 334–339. doi:10.1016/j.envres.2008.06.006
41. Channa, K., Odland, J. Ø., Kootbodien, T., Theodorou, P., Naik, I., Sandanger, T. M., & Röllin, H. B. (2013). Differences in prenatal exposure to mercury in South African communities residing along the Indian Ocean. Science of The Total Environment, 463-464, 11–19.doi:10.1016/j.scitotenv.2013.05.055
42. Chen, G., Chen, X., Yan, C., Wu, X., & Zeng, G. (2014). Surveying Mercury Levels in Hair, Blood and Urine of under 7-Year Old Children from a Coastal City in China. International Journal of Environmental Research and Public Health, 11(11), 12029–12041.doi:10.3390/ijerph111112029
43. Choi, J., Chang, J., Hong, J., Shin, S., Park, J., & Oh, S. (2017). Low-Level Toxic Metal Exposure in Healthy Weaning-Age Infants: Association with Growth, Dietary Intake, and Iron Deficiency. International Journal of Environmental Research and Public Health, 14(4), 388.doi:10.3390/ijerph14040388
44. Chojnacka, K., Zielińska, A., Górecka, H., Dobrzański, Z., & Górecki, H. (2010). Reference values for hair minerals of Polish students. Environmental Toxicology and Pharmacology, 29(3), 314–319.doi:10.1016/j.etap.2010.03.01
45. Christensen, K. Y., Thompson, B. A., Werner, M., Malecki, K., Imm, P., & Anderson, H. A. (2016). Levels of persistent contaminants in relation to fish consumption among older male anglers in Wisconsin. International Journal of Hygiene and Environmental Health, 219(2), 184–194. doi:10.1016/j.ijheh.2015.11.001
46. Corbett, C. E. P., El Khouri, M., Costa, A. N., Gyuricza, J. V., Corbett, J. F., Frizzarini, R., … Pinheiro, M. da C. (2007). Health Evaluation of Gold Miners Living in a Mercury-Contaminated Village in Serra Pelada, Pará, Brazil. Archives of Environmental & Occupational Health, 62(3), 121–128.doi:10.3200/aeoh.62.3.121-128
47. Cortes-Maramba, N., Reyes, J. P., Francisco-Rivera, A. T., Akagi, H., Sunio, R., & Panganiban, L. C. (2006). Health and environmental assessment of mercury exposure in a gold mining community in Western Mindanao, Philippines. Journal of Environmental Management, 81(2), 126–134.doi:10.1016/j.jenvman.2006.01.019
48. Curren, M. S., Liang, C. L., Davis, K., Kandola, K., Brewster, J., Potyrala, M., & Chan, H. M. (2015). Assessing determinants of maternal blood concentrations for persistent organic pollutants and metals in the eastern and western Canadian Arctic. Science of The Total Environment, 527-528, 150–158.doi:10.1016/j.scitotenv.2015.04.079
49. De Lourdes Soto-Ríos, M., Rothenberg, S. J., Juárez-Pérez, C. A., & Aguilar-Madrid, G. (2010). Variability of Mercury in Urine Among Mexican Women Residing in a Mining Area. Journal of Occupational and Environmental Medicine, 52(1), 62–66.doi:10.1097/jom.0b013e3181c75469
50. Decharat, S. (2012). Mercury Exposure among Garbage Workers in Southern Thailand. Safety and Health at Work, 3(4), 268–277.doi:10.5491/shaw.2012.3.4.268
51. Decharat, S., Phethuayluk, P., Maneelok, S., & Thepaksorn, P. (2014). Determination of Mercury Exposure among Dental Health Workers in Nakhon Si Thammarat Province, Thailand. Journal of Toxicology, 2014, 1–8. doi:10.1155/2014/401012
52. Deng C, Xie H, Ye X, et al. Mercury risk assessment combining internal and external exposure methods

for a population living near a municipal solid waste incinerator[J]. Environmental Pollution, 2016: 1060-1068.

53. Díez, S., Esbrí, J. M., Tobias, A., Higueras, P., & Martínez-Coronado, A. (2011). Determinants of exposure to mercury in hair from inhabitants of the largest mercury mine in the world. Chemosphere, 84(5), 571–577.doi:10.1016/j.chemosphere.2011.03.065
54. Díez, S., Montuori, P., Pagano, A., Sarnacchiaro, P., Bayona, J. M., & Triassi, M. (2008). Hair mercury levels in an urban population from southern Italy: Fish consumption as a determinant of exposure. Environment International, 34(2), 162–167.doi:10.1016/j.envint.2007.07.015
55. Ding, G., Cui, C., Chen, L., Gao, Y., Zhou, Y., Shi, R., & Tian, Y. (2013). Prenatal low-level mercury exposure and neonatal anthropometry in rural northern China. Chemosphere, 92(9), 1085–1089.doi:10.1016/j.chemosphere.2013.01.045
56. Dong, Z., Bank, M., & Spengler, J. (2015). Assessing Metal Exposures in a Community near a Cement Plant in the Northeast U.S. International Journal of Environmental Research and Public Health, 12(1), 952–969.doi:10.3390/ijerph120100952
57. Dong, Z., Jim, R. C., Hatley, E. L., Backus, A. S. N., Shine, J. P., Spengler, J. D., & Schaider, L. A. (2015). A longitudinal study of mercury exposure associated with consumption of freshwater fish from a reservoir in rural south central USA. Environmental Research, 136, 155–162. doi:10.1016/j.envres.2014.09.029
58. Dórea, J. G., de Souza, J. R., Rodrigues, P., Ferrari, Í., & Barbosa, A. C. (2005). Hair mercury (signature of fish consumption) and cardiovascular risk in Munduruku and Kayabi Indians of Amazonia. Environmental Research, 97(2), 209–219.doi:10.1016/j.envres.2004.04.007
59. Drescher, O., Dewailly, E., Diorio, C., Ouellet, N., Sidi, E. A. L., Abdous, B., … Ayotte, P. (2014). Methylmercury exposure, PON1 gene variants and serum paraoxonase activity in Eastern James Bay Cree adults. Journal of Exposure Science & Environmental Epidemiology, 24(6), 608–614.doi:10.1038/jes.2013.96
60. Du, B., Li, P., Feng, X., Qiu, G., Zhou, J., & Maurice, L. (2016). Mercury Exposure in Children of the Wanshan Mercury Mining Area, Guizhou, China. International Journal of Environmental Research and Public Health, 13(11), 1107. doi:10.3390/ijerph13111107
61. Du, B., Zhou, J., & Zhou, J. (2017). Selenium status of children in Kashin–Beck disease endemic areas in Shaanxi, China: assessment with mercury. Environmental Geochemistry and Health, 40(2), 903–913. doi:10.1007/s10653-017-0033-4
62. Egorov, A. I., Ilchenko, I. N., Lyapunov, S. M., Marochkina, E. V., Okina, O. I., Ermolaev, B. V., & Karamysheva, T. V. (2014). [Application of a standardized-human biomonitoring methodology to assess prenatal exposure to mercury].. Gigiena i sanitariia,.
63. Elenge, M. M., Aubry, J. C., Jacob, L., & De Brouwer, C. (2011). Heavy metal in hair samples of 109 non-industrial (miners) population in Katanga. Cahiers d'études et de recherches francophones/Santé, 21(1), 41-46.
64. Elhamri, H., Idrissi, L., Coquery, M., Azemard, S., Abidi, A. E., Benlemlih, M., … Cubadda, F. (2007). Hair mercury levels in relation to fish consumption in a community of the Moroccan

Mediterranean coast. Food Additives & Contaminants, 24(11), 1236–1246.doi:10.1080/02652030701329611

65. Eom, S.-Y., Lee, Y.-S., Lee, S.-G., Seo, M.-N., Choi, B.-S., Kim, Y.-D., … Park, J.-D. (2018). Lead, Mercury, and Cadmium Exposure in the Korean General Population. Journal of Korean Medical Science, 33(2).doi:10.3346/jkms.2018.33.e9
66. Erdinger, L., Eckl, P., Ingel, F., Khussainova, S., Utegenova, E., Mann, V., & Gabrio, T. (2004). The Aral Sea disaster – human biomonitoring of Hg, As, HCB, DDE, and PCBs in children living in Aralsk and Akchi, Kazakhstan. International Journal of Hygiene and Environmental Health, 207(6), 541–547.doi:10.1078/1438-4639-00325
67. Fakour, H., Esmaili-Sari, A., & Zayeri, F. (2010). Mercury exposure assessment in Iranian women's hair of a port town with respect to fish consumption and amalgam fillings. Science of The Total Environment, 408(7), 1538–1543.doi:10.1016/j.scitotenv.2010.01.008
68. Ferré-Huguet, N., Nadal, M., Schuhmacher, M., & Domingo, J. L. (2008). Monitoring Metals in Blood and Hair of the Population Living Near a Hazardous Waste Incinerator: Temporal Trend. Biological Trace Element Research, 128(3), 191–199.doi:10.1007/s12011-008-8274-9
69. Fløtre, C. H., Varsi, K., Helm, T., Bolann, B., & Bjørke-Monsen, A.-L. (2017). Predictors of mercury, lead, cadmium and antimony status in Norwegian never-pregnant women of fertile age. PLOS ONE, 12(12), e0189169.doi:10.1371/journal.pone.0189169
70. Fonseca, M. de F., De Souza Hacon, S., Grandjean, P., Choi, A. L., & Bastos, W. R. (2014). Iron status as a covariate in methylmercury-associated neurotoxicity risk. Chemosphere, 100, 89–96.doi:10.1016/j.chemosphere.2013.12.053
71. Fontaine, J., Dewailly, É., Benedetti, J.-L., Pereg, D., Ayotte, P., & Déry, S. (2008). Re-evaluation of blood mercury, lead and cadmium concentrations in the Inuit population of Nunavik (Québec): a cross-sectional study. Environmental Health, 7(1). doi:10.1186/1476-069x-7-25
72. Forde, M. S., Dewailly, E., Robertson, L., Laouan Sidi, E. A., Côté, S., Sandy, L., … Ayotte, P. (2014). Mercury and lead blood concentrations in pregnant women from 10 caribbean countries. Environ. Sci.: Processes Impacts, 16(9), 2184–2190. doi:10.1039/c4em00239c
73. Foster, W. G., Cheung, A. P., Davis, K., Graves, G., Jarrell, J., Leblanc, A., … Van Oostdam, J. (2012). Circulating metals and persistent organic pollutant concentrations in Canadian and non-Canadian born primiparous women from five Canadian centres: Results of a pilot biomonitoring study. Science of The Total Environment, 435-436, 326–336.doi:10.1016/j.scitotenv.2012.06.070
74. Franken, C., Koppen, G., Lambrechts, N., Govarts, E., Bruckers, L., Den Hond, E., … Schoeters, G. (2017). Environmental exposure to human carcinogens in teenagers and the association with DNA damage. Environmental Research, 152, 165–174.doi:10.1016/j.envres.2016.10.012
75. Fujimura, M., Matsuyama, A., Harvard, J.-P., Bourdineaud, J.-P., & Nakamura, K. (2011). Mercury Contamination in Humans in Upper Maroni, French Guiana Between 2004 and 2009. Bulletin of Environmental Contamination and Toxicology, 88(2), 135–139. doi:10.1007/s00128-011-0497-3
76. García-Esquinas, E., Pérez-Gómez, B., Fernández-Navarro, P., Fernández, M. A., de Paz, C., Pérez-Meixeira, A. M., … Aragonés, N. (2013). Lead, mercury and cadmium in umbilical cord blood and its association with parental epidemiological variables and birth factors. BMC Public Health, 13(1).doi:10.1186/1471-2458-13-841

77. Gatti, M. G., Bechtold, P., Campo, L., Barbieri, G., Quattrini, G., Ranzi, A., … Fustinoni, S. (2017). Human biomonitoring of polycyclic aromatic hydrocarbonsand metals in the general population residing near the municipal solid waste incinerator of Modena, Italy. Chemosphere, 186, 546–557.doi:10.1016/j.chemosphere.2017.07.122
78. Gerhardsson, L., & Lundh, T. (2010). Metal concentrations in blood and hair in pregnant females in southern Sweden.. Journal of Environmental Health, 72(6).
79. Gibb, H., Haver, C., Kozlov, K., Centeno, J. A., Jurgenson, V., Kolker, A., … Xu, H. (2011). Biomarkers of Mercury Exposure in Two Eastern Ukraine Cities. Journal of Occupational and Environmental Hygiene, 8(4), 187–193.doi:10.1080/15459624.2011.556984
80. Gibb, H., O'Leary, K. G., Sarkar, S. K., Wang, J., Liguori, L., Rainis, H., … Chatterjee, M. (2016). Hair mercury concentrations in residents of Sundarban and Calcutta, India. Environmental Research, 150, 616–621.doi:10.1016/j.envres.2016.03.028
81. Gobeille, A. K., Morland, K. B., Bopp, R. F., Godbold, J. H., & Landrigan, P. J. (2006). Body burdens of mercury in lower Hudson River area anglers. Environmental Research, 101(2), 205–212. doi:10.1016/j.envres.2005.08.017
82. Goodrich, J. M., Chou, H.-N., Gruninger, S. E., Franzblau, A., & Basu, N. (2015). Exposures of dental professionals to elemental mercury and methylmercury. Journal of Exposure Science & Environmental Epidemiology, 26(1), 78–85. doi:10.1038/jes.2015.52
83. Goodrich, J. M., Wang, Y., Gillespie, B., Werner, R., Franzblau, A., & Basu, N. (2011). Glutathione enzyme and selenoprotein polymorphisms associate with mercury biomarker levels in Michigan dental professionals. Toxicology and Applied Pharmacology, 257(2), 301–308.doi:10.1016/j.taap.2011.09.014
84. GUNDACKER, C., KOMARNICKI, G., JAGIELLO, P., GENCIKOVA, A., DAHMEN, N., WITTMANN, K., & GENCIK, M. (2007). Glutathione-S-transferase polymorphism, metallothionein expression, and mercury levels among students in Austria. Science of The Total Environment, 385(1-3), 37–47.doi:10.1016/j.scitotenv.2007.07.033
85. Guo, B.-Q., Cai, S.-Z., Guo, J.-L., Xu, J., Wu, W., Li, H., … Lü, H.-D. (2013). Levels of prenatal mercury exposure and their relationships to neonatal anthropometry in Wujiang City, China. Environmental Pollution, 182, 184–189.doi:10.1016/j.envpol.2013.07.023
86. Gustin, K., Tofail, F., Mehrin, F., Levi, M., Vahter, M., & Kippler, M. (2017). Methylmercury exposure and cognitive abilities and behavior at 10 years of age. Environment International, 102, 97–105. doi:10.1016/j.envint.2017.02.004
87. Ha, M., Kwon, H.-J., Lim, M.-H., Jee, Y.-K., Hong, Y.-C., Leem, J.-H., … Jo, S.-J. (2009). Low blood levels of lead and mercury and symptoms of attention deficit hyperactivity in children: A report of the children's health and environment research (CHEER). NeuroToxicology, 30(1), 31–36.doi:10.1016/j.neuro.2008.11.011
88. Habiba, G., Abebe, G., Bravo, A. G., Ermias, D., Staffan, Ǻ., & Bishop, K. (2016). Mercury Human Exposure in Populations Living Around Lake Tana (Ethiopia). Biological Trace Element Research, 175(2), 237–243.doi:10.1007/s12011-016-0745-9
89. Hacon, S., Dórea, J., Fonseca, M., Oliveira, B., Mourão, D., Ruiz, C., … Bastos, W. (2014). The Influence of Changes in Lifestyle and Mercury Exposure in Riverine Populations of the Madeira River (Amazon Basin) near a Hydroelectric Project. International Journal of Environmental Research and Public Health,

11(3), 2437–2455.doi:10.3390/ijerph110302437

90. Hansen, S., Nieboer, E., Sandanger, T. M., Wilsgaard, T., Thomassen, Y., Veyhe, A. S., & Odland, J. Ø. (2011). Changes in maternal blood concentrations of selected essential and toxic elements during and after pregnancy. Journal of Environmental Monitoring, 13(8), 2143.doi:10.1039/c1em10051c
91. Hinwood, A. L., Callan, A. C., Ramalingam, M., Boyce, M., Heyworth, J., McCafferty, P., & Odland, J. Ø. (2013). Cadmium, lead and mercury exposure in non smoking pregnant women. Environmental Research, 126, 118–124.doi:10.1016/j.envres.2013.07.005
92. Hohenblum, P., Steinbichl, P., Raffesberg, W., Weiss, S., Moche, W., Vallant, B., … Hutter, H.-P. (2012). Pollution gets personal! A first population-based human biomonitoring study in Austria. International Journal of Hygiene and Environmental Health, 215(2), 176–179.doi:10.1016/j.ijheh.2011.08.015
93. Hong C, Yu X, Liu J, et al. Low-level methylmercury exposure through rice ingestion in a cohort of pregnant mothers in rural China[J]. Environmental Research, 2016: 519-527.
94. Hsiao, H.-W., Ullrich, S. M., & Tanton, T. W. (2011). Burdens of mercury in residents of Temirtau, Kazakhstan. Science of The Total Environment, 409(11), 2272–2280.doi:10.1016/j.scitotenv.2009.12.040
95. Hsu, C.-S., Liu, P.-L., Chien, L.-C., Chou, S.-Y., & Han, B.-C. (2006). Mercury concentration and fish consumption in Taiwanese pregnant women. BJOG: An International Journal of Obstetrics & Gynaecology, 114(1), 81–85.doi:10.1111/j.1471-0528.2006.01142.x
96. Huang, S.-H., Weng, K.-P., Ger, L.-P., Liou, H. H., Lin, C.-C., Wang, C.-C., … Wu, M.-T. (2017). Influence of seafood and vitamin supplementation on maternal and umbilical cord blood mercury concentration. Journal of the Chinese Medical Association, 80(5), 307–312. doi:10.1016/j.jcma.2016.11.005
97. Huang, S.-H., Weng, K.-P., Lin, C.-C., Wang, C.-C., Lee, C. T.-C., Ger, L.-P., & Wu, M.-T. (2017). Maternal and umbilical cord blood levels of mercury, manganese, iron, and copper in southern Taiwan: A cross-sectional study. Journal of the Chinese Medical Association, 80(7), 442–451. doi:10.1016/j.jcma.2016.06.007
98. Ilmiawati, C., Yoshida, T., Itoh, T., Nakagi, Y., Saijo, Y., Sugioka, Y., … Kayama, F. (2014). Biomonitoring of mercury, cadmium, and lead exposure in Japanese children: a cross-sectional study. Environmental Health and Preventive Medicine, 20(1), 18–27.doi:10.1007/s12199-014-0416-4
99. Indriati Arifin, Y., Sakakibara, M., & Sera, K. (2017). Heavy metals concentrations in scalp hairs of ASGM miners and inhabitants of the Gorontalo Utara regency. IOP Conference Series: Earth and Environmental Science, 71, 012028.doi:10.1088/1755-1315/71/1/012028
100. Innis, S. M., Palaty, J., Vaghri, Z., & Lockitch, G. (2006). Increased levels of mercury associated with high fish intakes among children from Vancouver, Canada. The Journal of Pediatrics, 148(6), 759–763.doi:10.1016/j.jpeds.2006.02.001
101. Jackson, L. W., Howards, P. P., Wactawski-Wende, J., & Schisterman, E. F. (2011). The association between cadmium, lead and mercury blood levels and reproductive hormones among healthy, premenopausal women. Human Reproduction, 26(10), 2887–2895.doi:10.1093/humrep/der250
102. Jarosinska, D., Barregård, L., Biesiada, M., Muszynska-Graca, M., Dabkowska, B., Denby, B., … Zielonka, U. (2006). Urinary mercury in adults in Poland living near a chloralkali plant. Science of The Total Environment, 368(1), 335–343.doi:10.1016/j.scitotenv.2005.09.069

103. Jarosińska, D., Horvat, M., Sällsten, G., Mazzolai, B., Dąbkowska, B., Prokopowicz, A., … Barregård, L. (2008). Urinary mercury and biomarkers of early renal dysfunction in environmentally and occupationally exposed adults: A three-country study. Environmental Research, 108(2), 224–232. doi:10.1016/j.envres.2008.06.005
104. Jeppesen, C., Valera, B., Nielsen, N. O., Bjerregaard, P., & Jørgensen, M. E. (2015). Association between whole blood mercury and glucose intolerance among adult Inuit in Greenland. Environmental Research, 143, 192–197.doi:10.1016/j.envres.2015.10.013
105. Jin, L., Liu, J., Ye, B., & Ren, A. (2014). Concentrations of selected heavy metals in maternal blood and associated factors in rural areas in Shanxi Province, China. Environment International, 66, 157–164.doi:10.1016/j.envint.2014.01.016
106. Jose, A., & Ray, J. G. (2018). Toxic heavy metals in human blood in relation to certain food and environmental samples in Kerala, South India. Environmental Science and Pollution Research, 25(8), 7946–7953. doi:10.1007/s11356-017-1112-x
107. Julander, A., Lundgren, L., Skare, L., Grandér, M., Palm, B., Vahter, M., & Lidén, C. (2014). Formal recycling of e-waste leads to increased exposure to toxic metals: An occupational exposure study from Sweden. Environment International, 73, 243–251.doi:10.1016/j.envint.2014.07.006
108. Karimi, R., Silbernagel, S., Fisher, N. S., & Meliker, J. R. (2014). Elevated blood Hg at recommended seafood consumption rates in adult seafood consumers. International Journal of Hygiene and Environmental Health, 217(7), 758–764.doi:10.1016/j.ijheh.2014.03.007
109. Karouna-Renier, N. K., Ranga Rao, K., Lanza, J. J., Rivers, S. D., Wilson, P. A., Hodges, D. K., … Ross, G. T. (2008). Mercury levels and fish consumption practices in women of child-bearing age in the Florida Panhandle. Environmental Research, 108(3), 320–326. doi:10.1016/j.envres.2008.08.005
110. Khoury, E. D. T., Souza, G. da S., da Costa, C. A., de Araújo, A. A. K., de Oliveira, C. S. B., Silveira, L. C. de L., & Pinheiro, M. da C. N. (2015). Somatosensory Psychophysical Losses in Inhabitants of Riverside Communities of the Tapajós River Basin, Amazon, Brazil: Exposure to Methylmercury Is Possibly Involved. PLOS ONE, 10(12), e0144625.doi:10.1371/journal.pone.0144625
111. Kim, D. S., Kim, J. H., Yang, W.-H., Moon, J. S., & Son, B. S. (2010). Biomonitoring of urinary mercury in Korean school children. Molecular & Cellular Toxicology, 6(4), 351–358.doi:10.1007/s13273-010-0047-9
112. Kim, D.-S., Kwon, Y. M., Chung, H.-U., Choi, K. H., Oh, H. J., & Lee, E.-H. (2012). Mercury exposure and color vision loss of some Koreans in a fishery area. Molecular & Cellular Toxicology, 8(4), 407–412. doi:10.1007/s13273-012-0050-4
113. Kim, E. H., Kim, I. K., Kwon, J. Y., Kim, S. W., & Park, Y. W. (2006). The Effect of Fish Consumption on Blood Mercury Levels of Pregnant Women. Yonsei Medical Journal, 47(5), 626.doi:10.3349/ymj.2006.47.5.626
114. Kim, H.-J., Lim, H.-S., Lee, K.-R., Choi, M.-H., Kang, N. M., Lee, C. H., … Park, H.-K. (2017). Determination of Trace Metal Levels in the General Population of Korea. International Journal of Environmental Research and Public Health, 14(7), 702.doi:10.3390/ijerph14070702
115. Kim, J. H., Lee, S. J., Kim, S. Y., Choi, G., Lee, J. J., Kim, H.-J., … Choi, S. ran. (2016). Association of food consumption during pregnancy with mercury and lead levels in cord blood. Science of The Total Environment, 563-564, 118–124.doi:10.1016/j.scitotenv.2016.04.082

116. Kim, N.-Y., Ahn, S.-J., Ryu, D.-Y., Choi, B.-S., Kim, H., Yu, I.-J., & Park, J.-D. (2012). Effect of lifestyles on the blood mercury level in Korean adults. Human & Experimental Toxicology, 32(6), 591–599. doi:10.1177/0960327112467041
117. Kim, S. A., Jeon, C. K., & Paek, D. M. (2008). Hair mercury concentrations of children and mothers in Korea: Implication for exposure and evaluation. Science of The Total Environment, 402(1), 36–42.doi:10.1016/j.scitotenv.2008.04.010
118. Kim, Y.-M., Chung, J.-Y., An, H., Park, S., Kim, B.-G., Bae, J., … Hong, Y.-S. (2015). Biomonitoring of Lead, Cadmium, Total Mercury, and Methylmercury Levels in Maternal Blood and in Umbilical Cord Blood at Birth in South Korea. International Journal of Environmental Research and Public Health, 12(10), 13482–13493.doi:10.3390/ijerph121013482
119. King, K. E., Darrah, T. H., Money, E., Meentemeyer, R., Maguire, R. L., Nye, M. D., … Hoyo, C. (2015). Geographic clustering of elevated blood heavy metal levels in pregnant women. BMC Public Health, 15(1).doi:10.1186/s12889-015-2379-9
120. Knobeloch, L., Gliori, G., & Anderson, H. (2007). Assessment of methylmercury exposure in Wisconsin. Environmental Research, 103(2), 205–210.doi:10.1016/j.envres.2006.05.012
121. Knobeloch, L., Tomasallo, C., & Anderson, H. A. (2011). Biomonitoring as an intervention against methylmercury exposure.. Public Health Reports, 126(4), 568-574.
122. Kobal, A. B., Snoj Tratnik, J., Mazej, D., Fajon, V., Gibičar, D., Miklavčič, A., … Horvat, M. (2017). Exposure to mercury in susceptible population groups living in the former mercury mining town of Idrija, Slovenia. Environmental Research, 152, 434–445. doi:10.1016/j.envres.2016.06.037
123. Kowalski, R., & Wiercinski, J. (2007). Determination of Total Mercury Concentration in Hair of Lubartów-Area Citizens (Lublin Region, Poland). Polish Journal of Environmental Studies, 16(1), 75-79.
124. Kuno, R., Roquetti, M. H., Becker, K., Seiwert, M., & Gouveia, N. (2013). Reference values for lead, cadmium and mercury in the blood of adults from the metropolitan area of Sao Paulo, Brazil. International Journal of Hygiene and Environmental Health, 216(3), 243–249.doi:10.1016/j.ijheh.2012.05.010
125. Kusanagi, E., Takamura, H., Chen, S.-J., Adachi, M., & Hoshi, N. (2018). Children's Hair Mercury Concentrations and Seafood Consumption in Five Regions of Japan. Archives of Environmental Contamination and Toxicology, 74(2), 259–272. doi:10.1007/s00244-017-0502-x
126. Laamech, J., Bernard, A., Dumont, X., Benazzouz, B., & Lyoussi, B. (2014). Blood lead, cadmium and mercury among children from urban, industrial and rural areas of Fez Boulemane Region (Morocco): Relevant factors and early renal effects. International Journal of Occupational Medicine and Environmental Health, 27(4).doi:10.2478/s13382-014-0275-7
127. Latshaw, M. W., Glass, T., Parsons, P., Hidalgo, J., & Schwartz, B. (2006). Predictors of Blood Mercury Levels in Older Urban Residents. Journal of Occupational and Environmental Medicine, 48(7), 715–722.doi:10.1097/01.jom.0000205208.85800.70
128. Lee, C. S., Lim, Y. W., Kim, H. H., Yang, J. Y., & Shin, D. C. (2011). Exposure to heavy metals in blood and risk perception of the population living in the vicinity of municipal waste incinerators in Korea. Environmental Science and Pollution Research, 19(5), 1629–1639. doi:10.1007/s11356-011-0677-z
129. Lee, J. W., Lee, C. K., Moon, C. S., Choi, I. J., Lee, K. J., Yi, S.-M., … Lee, J. H. (2012). Korea National Survey for Environmental Pollutants in the Human Body 2008: Heavy metals in the blood or urine of the Korean population. International Journal of Hygiene and Environmental Health, 215(4), 449–

457.doi:10.1016/j.ijheh.2012.01.002
130. Lee, M.-R., Lim, Y.-H., Lee, B.-E., & Hong, Y.-C. (2017). Blood mercury concentrations are associated with decline in liver function in an elderly population: a panel study. Environmental Health, 16(1).doi:10.1186/s12940-017-0228-2
131. Lee, S., Shin, M., Hong, Y.-C., & Kim, J. H. (2017). Temporal variability of blood lead, mercury, and cadmium levels in elderly panel study (2008–2014). International Journal of Hygiene and Environmental Health, 220(2), 407–414. doi:10.1016/j.ijheh.2016.11.014
132. Legrand, M., Arp, P., Ritchie, C., & Chan, H. M. (2005). Mercury exposure in two coastal communities of the Bay of Fundy, Canada. Environmental Research, 98(1), 14–21.doi:10.1016/j.envres.2004.07.006
133. Levy, L. S., Jones, K., Cocker, J., Assem, F. L., & Capleton, A. C. (2007). Background levels of key biomarkers of chemical exposure within the UK general population – Pilot study. International Journal of Hygiene and Environmental Health, 210(3-4), 387–391. doi:10.1016/j.ijheh.2007.01.032
134. Li, M., Wu, M., Xu, J., Du, J., & Yan, C. (2014). Body Burden of Hg in Different Bio-Samples of Mothers in Shenyang City, China. PLoS ONE, 9(5), e98121.doi:10.1371/journal.pone.0098121
135. Li, P., Feng, X., Chan, H.-M., Zhang, X., & Du, B. (2015). Human Body Burden and Dietary Methylmercury Intake: The Relationship in a Rice-Consuming Population. Environmental Science & Technology, 49(16), 9682–9689.doi:10.1021/acs.est.5b00195
136. Li, P., Feng, X., Qiu, G., Shang, L., & Li, G. (2009). Human hair mercury levels in the Wanshan mercury mining area, Guizhou Province, China. Environmental Geochemistry and Health, 31(6), 683–691.doi:10.1007/s10653-008-9246-x
137. Li, P., Feng, X., Qiu, G., Shang, L., & Wang, S. (2008). Mercury exposure in the population from Wuchuan mercury mining area, Guizhou, China. Science of The Total Environment, 395(2-3), 72–79. doi:10.1016/j.scitotenv.2008.02.006
138. Li, Y., Zhang, B., Yang, L., & Li, H. (2012). Blood mercury concentration among residents of a historic mercury mine and possible effects on renal function: a cross-sectional study in southwestern China. Environmental Monitoring and Assessment, 185(4), 3049–3055. doi:10.1007/s10661-012-2772-0
139. Li, Y., Zhang, X., Yang, L., & Li, H. (2012). Levels of Cd, Pb, As, Hg, and Se in Hair of Residents Living in Villages Around Fenghuang Polymetallic Mine, Southwestern China. Bulletin of Environmental Contamination and Toxicology, 89(1), 125–128.doi:10.1007/s00128-012-0650-7
140. Liang, G., Pan, L., & Liu, X. (2017). Assessment of Typical Heavy Metals in Human Hair of Different Age Groups and Foodstuffs in Beijing, China. International Journal of Environmental Research and Public Health, 14(8), 914.doi:10.3390/ijerph14080914
141. Liang, P., Feng, X., Zhang, C., Zhang, J., Cao, Y., You, Q., … Wu, S.-C. (2015). Human exposure to mercury in a compact fluorescent lamp manufacturing area: By food (rice and fish) consumption and occupational exposure. Environmental Pollution, 198, 126–132.doi:10.1016/j.envpol.2014.12.036
142. Liberda, E. N., Tsuji, L. J. S., Martin, I. D., Ayotte, P., Dewailly, E., & Nieboer, E. (2014). The complexity of hair/blood mercury concentration ratios and its implications. Environmental Research, 134, 286–294.doi:10.1016/j.envres.2014.08.007
143. Lim, S., Chung, H.-U., & Paek, D. (2010). Low dose mercury and heart rate variability among community residents nearby to an industrial complex in Korea. NeuroToxicology, 31(1), 10–16.doi:10.1016/j.neuro.2009.10.001

144. Lincoln, R. A., Shine, J. P., Chesney, E. J., Vorhees, D. J., Grandjean, P., & Senn, D. B. (2010). Fish Consumption and Mercury Exposure among Louisiana Recreational Anglers. Environmental Health Perspectives, 119(2), 245–251.doi:10.1289/ehp.1002609
145. Liu, J.-L., Xu, X.-R., Yu, S., Cheng, H., Peng, J.-X., Hong, Y.-G., & Feng, X.-B. (2014). Mercury contamination in fish and human hair from Hainan Island, South China Sea: Implication for human exposure. Environmental Research, 135, 42–47.doi:10.1016/j.envres.2014.08.023
146. Liu, J.-L., Xu, X.-R., Yu, S., Cheng, H., Peng, J.-X., Hong, Y.-G., & Feng, X.-B. (2014). Mercury contamination in fish and human hair from Hainan Island, South China Sea: Implication for human exposure. Environmental Research, 135, 42–47.doi:10.1016/j.envres.2014.08.023
147. Luo, R., Zhuo, X., & Ma, D. (2014). Determination of 33 elements in scalp hair samples from inhabitants of a mountain village of Tonglu city, China. Ecotoxicology and Environmental Safety, 104, 215–219.doi:10.1016/j.ecoenv.2014.03.006
148. Marcinek-Jacel, M., Albińska, J., Pawlaczyk, A., & Szynkowska, M. I. (2017). The impact of demographic factors, behaviors and environmental exposure to mercury content in the hair of the population living in the region of Lodz (central Poland). Environmental Toxicology and Pharmacology, 55, 196–201.doi:10.1016/j.etap.2017.09.001
149. Marinho, J. S., Lima, M. O., Santos, E. C. de O., de Jesus, I. M., Pinheiro, M. da C. N., Alves, C. N., & Muller, R. C. S. (2014). Mercury Speciation in Hair of Children in Three Communities of the Amazon, Brazil. BioMed Research International, 2014, 1–9.doi:10.1155/2014/945963
150. Marques, R. C., Dórea, J. G., Leão, R. S., dos Santos, V. G., Bueno, L., Marques, R. C., … Guimarães, J. R. D. (2011). Role of Methylmercury Exposure (from Fish Consumption) on Growth and Neurodevelopment of Children Under 5 Years of Age Living in a Transitioning (Tin-Mining) Area of the Western Amazon, Brazil. Archives of Environmental Contamination and Toxicology, 62(2), 341–350.doi:10.1007/s00244-011-9697-4
151. Marques, R. C., Garrofe Dórea, J., Rodrigues Bastos, W., de Freitas Rebelo, M., de Freitas Fonseca, M., & Malm, O. (2007). Maternal mercury exposure and neuro-motor development in breastfed infants from Porto Velho (Amazon), Brazil. International Journal of Hygiene and Environmental Health, 210(1), 51–60.doi:10.1016/j.ijheh.2006.08.001
152. Masih, A., Taneja, A., & Singhvi, R. (2015). Exposure profiles of mercury in human hair at a terai belt of North India. Environmental Geochemistry and Health, 38(1), 145–156.doi:10.1007/s10653-015-9698-8
153. McKelvey, W., Gwynn, C., Jeffery, N., Kass, D., Thorpe, L., Garg, R., … Parsons, P. J. (2007). A Biomonitoring Study of Lead, Cadmium and Mercury in the Blood of New York City Adults. Environmental Health Perspectives.doi:10.1289/ehp.10056
154. Mezghani-Chaari, S., Hamza, A., & Hamza-Chaffai, A. (2010). Mercury contamination in human hair and some marine species from Sfax coasts of Tunisia: levels and risk assessment. Environmental Monitoring and Assessment, 180(1-4), 477–487.doi:10.1007/s10661-010-1800-1
155. Miklavčič, A., Cuderman, P., Mazej, D., Snoj Tratnik, J., Krsnik, M., Planinšek, P., … Horvat, M. (2011). Biomarkers of low-level mercury exposure through fish consumption in pregnant and lactating Slovenian women. Environmental Research, 111(8), 1201–1207. doi:10.1016/j.envres.2011.07.006
156. Miyake, Y., Tanaka, K., Yasutake, A., Sasaki, S., & Hirota, Y. (2011). Lack of association of mercury with risk of wheeze and eczema in Japanese children: The Osaka Maternal and Child Health Study.

Environmental Research, 111(8), 1180–1184.doi:10.1016/j.envres.2011.07.003
157. Miyashita, C., Sasaki, S., Ikeno, T., Araki, A., Ito, S., Kajiwara, J., … Kishi, R. (2015). Effects of in utero exposure to polychlorinated biphenyls, methylmercury, and polyunsaturated fatty acids on birth size. Science of The Total Environment, 533, 256–265.doi:10.1016/j.scitotenv.2015.06.108
158. Miyashita, C., Sasaki, S., Saijo, Y., Okada, E., Kobayashi, S., Baba, T., … Kishi, R. (2015). Demographic, behavioral, dietary, and socioeconomic characteristics related to persistent organic pollutants and mercury levels in pregnant women in Japan. Chemosphere, 133, 13–21.doi:10.1016/j.chemosphere.2015.02.062
159. Mocevic, E., Specht, I. O., Marott, J. L., Giwercman, A., Jönsson, B. A., Toft, G., … Peter Bonde, J. (2012). Environmental mercury exposure, semen quality and reproductive hormones in Greenlandic Inuit and European men: a cross-sectional study. Asian Journal of Andrology, 15(1), 97–104.doi:10.1038/aja.2012.121
160. Molina-Villalba, I., Lacasaña, M., Rodríguez-Barranco, M., Hernández, A. F., Gonzalez-Alzaga, B., Aguilar-Garduño, C., & Gil, F. (2015). Biomonitoring of arsenic, cadmium, lead, manganese and mercury in urine and hair of children living near mining and industrial areas. Chemosphere, 124, 83–91.doi:10.1016/j.chemosphere.2014.11.016
161. Moreno, M. E., Acosta-Saavedra, L. C., Meza-Figueroa, D., Vera, E., Cebrian, M. E., Ostrosky-Wegman, P., & Calderon-Aranda, E. S. (2010). Biomonitoring of metal in children living in a mine tailings zone in Southern Mexico: A pilot study. International Journal of Hygiene and Environmental Health, 213(4), 252–258.doi:10.1016/j.ijheh.2010.03.005
162. Motawei, S. M., & Gouda, H. E. (2015). Screening of Blood Levels of Mercury, Cadmium, and Copper in Pregnant Women in Dakahlia, Egypt: New Attention to an Old Problem. Biological Trace Element Research, 171(2), 308–314. doi:10.1007/s12011-015-0525-y
163. Murata, K., Sakamoto, M., Nakai, K., Dakeishi, M., Iwata, T., Liu, X.-J., & Satoh, H. (2005). Subclinical effects of prenatal methylmercury exposure on cardiac autonomic function in Japanese children. International Archives of Occupational and Environmental Health, 79(5), 379–386.doi:10.1007/s00420-005-0064-5
164. Murata, K., Sakamoto, M., Nakai, K., Weihe, P., Dakeishi, M., Iwata, T., … Satoh, H. (2004). Effects of methylmercury on neurodevelopment in Japanese children in relation to the Madeiran study. International Archives of Occupational and Environmental Health, 77(8), 571–579.doi:10.1007/s00420-004-0542-1
165. Nadal, M., Bocio, A., Schuhmacher, M., & Domingo, J. L. (2005). Monitoring Metals in the Population Living in the Vicinity of a Hazardous Waste Incinerator: Levels in Hair of School Children. Biological Trace Element Research, 104(3), 203–214.doi:10.1385/bter:104:3:203
166. Nakamura, M., Hachiya, N., Murata, K., Nakanishi, I., Kondo, T., Yasutake, A., … Sakamoto, M. (2014). Methylmercury exposure and neurological outcomes in Taiji residents accustomed to consuming whale meat. Environment International, 68, 25–32.doi:10.1016/j.envint.2014.03.005
167. Ni, W., Chen, Y., Huang, Y., Wang, X., Zhang, G., Luo, J., & Wu, K. (2014). Hair mercury concentrations and associated factors in an electronic waste recycling area, Guiyu, China. Environmental Research, 128, 84–91.doi:10.1016/j.envres.2013.10.005
168. Niane, B., Guédron, S., Moritz, R., Cosio, C., Ngom, P. M., Deverajan, N., … Poté, J. (2014). Human exposure to mercury in artisanal small-scale gold mining areas of Kedougou region, Senegal, as a function of occupational activity and fish consumption. Environmental Science and Pollution Research, 22(9),

7101–7111. doi:10.1007/s11356-014-3913-5

169. Nisse, C., Tagne-Fotso, R., Howsam, M., Richeval, C., Labat, L., & Leroyer, A. (2017). Blood and urinary levels of metals and metalloids in the general adult population of Northern France: The IMEPOGE study, 2008–2010. International Journal of Hygiene and Environmental Health, 220(2), 341–363. doi:10.1016/j.ijheh.2016.09.020
170. Nunes, E., Cavaco, A., & Carvalho, C. (2014). Exposure Assessment of Pregnant Portuguese Women to Methylmercury Through the Ingestion of Fish: Cross-Sectional Survey and Biomarker Validation. Journal of Toxicology and Environmental Health, Part A, 77(1-3), 133–142.doi:10.1080/15287394.2014.867200
171. Obeid, P. J., Fares, S. A., Farhat, G. N., El-Khoury, B., Nassif, R. M., El-Nakat, J., & Dhaini, H. R. (2017). Mercury health risk assessment among a young adult Lebanese population. Environmental Science and Pollution Research, 24(10), 9370–9378. doi:10.1007/s11356-017-8621-5
172. Obi, E., Okafor, C., Igwebe, A., Ebenebe, J., Afonne, O. J., Ifediata, F., … Basu, N. (2015). Elevated prenatal methylmercury exposure in Nigeria: Evidence from maternal and cord blood. Chemosphere, 119, 485–489.doi:10.1016/j.chemosphere.2014.07.038
173. Ojo, J. O., Oketayo, O. O., Adesanmi, C. A., Horvat, M., Mazej, D., & Tratnik, J. (2013). Influence of nutritional status on some toxic and essential elements in the blood of women exposed to vehicular pollution in Ile-Ife, Nigeria. Environmental Science and Pollution Research, 21(2), 1124–1132.doi:10.1007/s11356-013-1951-z
174. Okati, N., & Esmaili-sari, A. (2017). Hair mercury and risk assessment for consumption of contaminated seafood in residents from the coast of the Persian Gulf, Iran. Environmental Science and Pollution Research, 25(1), 639–657. doi:10.1007/s11356-017-0432-1
175. Okati, N., Sari, A. E., & Ghasempouri, S. M. (2012). Hair Mercury Concentrations of Lactating Mothers and Breastfed Infants in Iran (Fish Consumption and Mercury Exposure). Biological Trace Element Research, 149(2), 155–162.doi:10.1007/s12011-012-9424-7
176. Oliveira, R. C., Dórea, J. G., Bernardi, J. V. E., Bastos, W. R., Almeida, R., & Manzatto, Â. G. (2010). Fish consumption by traditional subsistence villagers of the Rio Madeira (Amazon): Impact on hair mercury. Annals of Human Biology, 37(5), 629–642.doi:10.3109/03014460903525177
177. Olivero-Verbel, J., Caballero-Gallardo, K., & Negrete-Marrugo, J. (2011). Relationship Between Localization of Gold Mining Areas and Hair Mercury Levels in People from Bolivar, North of Colombia. Biological Trace Element Research, 144(1-3), 118–132.doi:10.1007/s12011-011-9046-5
178. Olivero-Verbel, J., Carranza-Lopez, L., Caballero-Gallardo, K., Ripoll-Arboleda, A., & Muñoz-Sosa, D. (2016). Human exposure and risk assessment associated with mercury pollution in the Caqueta River, Colombian Amazon. Environmental Science and Pollution Research, 23(20), 20761–20771.doi:10.1007/s11356-016-7255-3
179. Ortiz-Roque, C. (2004). Mercury contamination in reproductive age women in a Caribbean island: Vieques. Journal of Epidemiology & Community Health, 58(9), 756–757.doi:10.1136/jech.2003.019224
180. Palacios-Torres, Y., Caballero-Gallardo, K., & Olivero-Verbel, J. (2018). Mercury pollution by gold mining in a global biodiversity hotspot, the Choco biogeographic region, Colombia. Chemosphere, 193, 421–430. doi:10.1016/j.chemosphere.2017.10.160
181. Pan S, Lin L, Zeng F, et al. Effects of lead, cadmium, arsenic, and mercury co-exposure on children's intelligence quotient in an industrialized area of southern China.[J]. Environmental Pollution, 2018: 47-54.

182. Park H, Kim D, Moon J, et al. Mercury Level in Hair of Primary School Children in Korea and China[J]. Molecular & Cellular Toxicology, 2008, 4(3): 235-245.
183. Park, G. I., Byun, Y. S., Joong Jeon, M., & Sakong, J. (2017). The associations between blood mercury levels and shark meat intake among workers in Gyeongsangbuk-do. Annals of Occupational and Environmental Medicine, 29(1). doi:10.1186/s40557-017-0185-9
184. Park, H., Lee, K., Moon, C.-S., Woo, K., Kang, T.-S., Chung, E.-K., & Son, B.-S. (2015). Simultaneous Exposure to Heavy Metals among Residents in the Industrial Complex: Korean National Cohort Study. International Journal of Environmental Research and Public Health, 12(6), 5905–5917.doi:10.3390/ijerph120605905
185. Park, H.-S., Shin, K.-O., & Kim, J.-S. (2007). Assessment of reference values for hair minerals of Korean preschool children. Biological Trace Element Research, 116(2), 119–130.doi:10.1007/bf02685925
186. Paruchuri, Y., Siuniak, A., Johnson, N., Levin, E., Mitchell, K., Goodrich, J. M., … Basu, N. (2010). Occupational and environmental mercury exposure among small-scale gold miners in the Talensi–Nabdam District of Ghana's Upper East region. Science of The Total Environment, 408(24), 6079–6085.doi:10.1016/j.scitotenv.2010.08.022
187. Passos, C. J. S., Da Silva, D. S., Lemire, M., Fillion, M., Guimarães, J. R. D., Lucotte, M., & Mergler, D. (2007). Daily mercury intake in fish-eating populations in the Brazilian Amazon. Journal of Exposure Science & Environmental Epidemiology, 18(1), 76–87. doi:10.1038/sj.jes.7500599
188. Passos, C. J. S., Mergler, D., Fillion, M., Lemire, M., Mertens, F., Guimarães, J. R. D., & Philibert, A. (2007). Epidemiologic confirmation that fruit consumption influences mercury exposure in riparian communities in the Brazilian Amazon. Environmental Research, 105(2), 183–193. doi:10.1016/j.envres.2007.01.012
189. Passos, C. J. S., Mergler, D., Lemire, M., Fillion, M., & Guimarães, J. R. D. (2007). Fish consumption and bioindicators of inorganic mercury exposure. Science of The Total Environment, 373(1), 68–76.doi:10.1016/j.scitotenv.2006.11.015
190. Pawlas, N., Strömberg, U., Carlberg, B., Cerna, M., Harari, F., Harari, R., … Bergdahl, I. (2013). Cadmium, mercury and lead in the blood of urban women in Croatia, the Czech Republic, Poland, Slovakia, Slovenia, Sweden, China, Ecuador and Morocco. International Journal of Occupational Medicine and Environmental Health, 26(1).doi:10.2478/s13382-013-0071-9
191. Pérez, R., Doménech, E., Conchado, A., Sanchez, A., Coscollà, C., & Yusà, V. (2018). Influence of diet in urinary levels of metals in a biomonitoring study of a child population of the Valencian region (Spain). Science of The Total Environment, 618, 1647–1657. doi:10.1016/j.scitotenv.2017.10.011
192. Perez-Maldonado, I. N., Ochoa-Martinez, A. C., Orta-Garcia, S. T., Ruiz-Vera, T., & Varela-Silva, J. A. (2017). Concentrations of Environmental Chemicals in Urine and Blood Samples of Children from San Luis Potosí, Mexico. Bulletin of Environmental Contamination and Toxicology, 99(2), 258–263. doi:10.1007/s00128-017-2130-6
193. Pino, A., Amato, A., Alimonti, A., Mattei, D., & Bocca, B. (2012). Human biomonitoring for metals in Italian urban adolescents: Data from Latium Region. International Journal of Hygiene and Environmental Health, 215(2), 185–190.doi:10.1016/j.ijheh.2011.07.015
194. Pirard, C., Compere, S., Firquet, K., & Charlier, C. (2018). The current environmental levels of endocrine disruptors (mercury, cadmium, organochlorine pesticides and PCBs) in a Belgian adult population and

their predictors of exposure. International Journal of Hygiene and Environmental Health, 221(2), 211–222. doi:10.1016/j.ijheh.2017.10.010

195. Polanska, K., Hanke, W., Sobala, W., Trzcinka-Ochocka, M., Ligocka, D., Brzeznicki, S., … Magnus, P. (2013). Developmental Effects of Exposures to Environmental Factors: The Polish Mother and Child Cohort Study. BioMed Research International, 2013, 1–11. doi:10.1155/2013/629716
196. Pouzaud, F., Ibbou, A., Blanchemanche, S., Grandjean, P., Krempf, M., Philippe, H.-J., & Verger, P. (2009). Use of advanced cluster analysis to characterize fish consumption patterns and methylmercury dietary exposures from fish and other sea foods among pregnant women. Journal of Exposure Science & Environmental Epidemiology, 20(1), 54–68.doi:10.1038/jes.2009.2
197. Quandt, S. A., Jones, B. T., Talton, J. W., Whalley, L. E., Galván, L., Vallejos, Q. M., … Arcury, T. A. (2010). Heavy metals exposures among Mexican farmworkers in eastern North Carolina. Environmental Research, 110(1), 83–88.doi:10.1016/j.envres.2009.09.007
198. Rahbar, M. H., Samms-Vaughan, M., Loveland, K. A., Ardjomand-Hessabi, M., Chen, Z., Bressler, J., … Boerwinkle, E. (2012). Seafood Consumption and Blood Mercury Concentrations in Jamaican Children With and Without Autism Spectrum Disorders. Neurotoxicity Research, 23(1), 22–38.doi:10.1007/s12640-012-9321-z
199. Rahbar, M., Samms-Vaughan, M., Dickerson, A., Hessabi, M., Bressler, J., Desai, C., … Boerwinkle, E. (2015). Concentration of Lead, Mercury, Cadmium, Aluminum, Arsenic and Manganese in Umbilical Cord Blood of Jamaican Newborns. International Journal of Environmental Research and Public Health, 12(5), 4481–4501.doi:10.3390/ijerph120504481
200. Rajaee, M., Long, R., Renne, E., & Basu, N. (2015). Mercury Exposure Assessment and Spatial Distribution in A Ghanaian Small-Scale Gold Mining Community. International Journal of Environmental Research and Public Health, 12(9), 10755–10782.doi:10.3390/ijerph120910755
201. Rajgopal T, Ravimohan H V, Mascarenhas P. Epidemiological surveillance of employees in a mercury thermometer plant: An occupational health study. Indian J Occup Environ Med 2006;10:11-8
202. Rambousková, J., Krsková, A., Slavíková, M., Čejchanová, M., & Černá, M. (2014). Blood levels of lead, cadmium, and mercury in the elderly living in institutionalized care in the Czech Republic. Experimental Gerontology, 58, 8–13.doi:10.1016/j.exger.2014.07.002
203. Ranzi, A., Fustinoni, S., Erspamer, L., Campo, L., Gatti, M. G., Bechtold, P., … Lauriola, P. (2013). Biomonitoring of the general population living near a modern solid waste incinerator: A pilot study in Modena, Italy. Environment International, 61, 88–97.doi:10.1016/j.envint.2013.09.008
204. Rice, D. C., Lincoln, R., Martha, J., Parker, L., Pote, K., Xing, S., & Smith, A. E. (2010). Concentration of metals in blood of Maine children 1–6 years old. Journal of Exposure Science & Environmental Epidemiology, 20(7), 634–643. doi:10.1038/jes.2010.42
205. Rignell-Hydbom, A., Axmon, A., Lundh, T., Jönsson, B. A., Tiido, T., & Spano, M. (2007). Dietary exposure to methyl mercury and PCB and the associations with semen parameters among Swedish fishermen. Environmental Health, 6(1). doi:10.1186/1476-069x-6-14
206. Röllin, H. B., Rudge, C. V. C., Thomassen, Y., Mathee, A., & Odland, J. Ø. (2009). Levels of toxic and essential metals in maternal and umbilical cord blood from selected areas of South Africa—results of a pilot study. Journal of Environmental Monitoring, 11(3), 618. doi:10.1039/b816236k

207. Rudge, C. V. C., Calderon, I. M. P., Rudge, M. V. C., Volpato, G., Silva, J. L. P., Duarte, G., … Odland, J. Ø. (2011). Toxic and essential elements in blood from delivering women in selected areas of São Paulo State, Brazil. J. Environ. Monit., 13(3), 563–571.doi:10.1039/c0em00570c
208. Salazar-Camacho, C., Salas-Moreno, M., Marrugo-Madrid, S., Marrugo-Negrete, J., & Díez, S. (2017). Dietary human exposure to mercury in two artisanal small-scale gold mining communities of northwestern Colombia. Environment International, 107, 47–54.doi:10.1016/j.envint.2017.06.011
209. Salehi, Z., & Esmaili-Sari, A. (2010). Hair mercury levels in pregnant women in Mahshahr, Iran: Fish consumption as a determinant of exposure. Science of The Total Environment, 408(20), 4848–4854.doi:10.1016/j.scitotenv.2010.06.027
210. Sanders, A. P., Flood, K., Chiang, S., Herring, A. H., Wolf, L., & Fry, R. C. (2012). Towards Prenatal Biomonitoring in North Carolina: Assessing Arsenic, Cadmium, Mercury, and Lead Levels in Pregnant Women. PLoS ONE, 7(3), e31354.doi:10.1371/journal.pone.0031354
211. Santos, E. O., Jesus, I. M. de, Câmara, V. de M., Brabo, E. da S., Jesus, M. I. de, Fayal, K. F., & Asmus, C. I. R. F. (2007). Correlation between blood mercury levels in mothers and newborns in Itaituba, Pará State, Brazil. Cadernos de Saúde Pública, 23(suppl 4), S622–S629.doi:10.1590/s0102-311x2007001600022
212. Schantz, S. L., Gardiner, J. C., Aguiar, A., Tang, X., Gasior, D. M., Sweeney, A. M., … Kostyniak, P. J. (2010). Contaminant profiles in Southeast Asian immigrants consuming fish from polluted waters in northeastern Wisconsin. Environmental Research, 110(1), 33–39.doi:10.1016/j.envres.2009.09.003
213. Schutzmeier, P., Berger, U., & Bose-O'Reilly, S. (2016). Gold Mining in Ecuador: A Cross-Sectional Assessment of Mercury in Urine and Medical Symptoms in Miners from Portovelo/Zaruma. International Journal of Environmental Research and Public Health, 14(1), 34.doi:10.3390/ijerph14010034
214. Sexton, K., Ryan, A. D., Adgate, J. L., Barr, D. B., & Needham, L. L. (2011). Biomarker Measurements of Concurrent Exposure to Multiple Environmental Chemicals and Chemical Classes in Children. Journal of Toxicology and Environmental Health, Part A, 74(14), 927–942.doi:10.1080/15287394.2011.573745
215. Simić, A., Hansen, A. F., Åsvold, B. O., Romundstad, P. R., Midthjell, K., Syversen, T., & Flaten, T. P. (2017). Trace element status in patients with type 2 diabetes in Norway: The HUNT3 Survey. Journal of Trace Elements in Medicine and Biology, 41, 91–98.doi:10.1016/j.jtemb.2017.03.001
216. Sirot, V., Guérin, T., Mauras, Y., Garraud, H., Volatier, J.-L., & Leblanc, J.-C. (2008). Methylmercury exposure assessment using dietary and biomarker data among frequent seafood consumers in France. Environmental Research, 107(1), 30–38.doi:10.1016/j.envres.2007.12.005
217. Skalny, A. V., Skalnaya, M. G., Tinkov, A. A., Serebryansky, E. P., Demidov, V. A., Lobanova, Y. N., … Nikonorov, A. A. (2015). Reference values of hair toxic trace elements content in occupationally non-exposed Russian population. Environmental Toxicology and Pharmacology, 40(1), 18–21.doi:10.1016/j.etap.2015.05.004
218. Son, J.-Y., Lee, J., Paek, D., & Lee, J.-T. (2009). Blood levels of lead, cadmium, and mercury in the Korean population: Results from the Second Korean National Human Exposure and Bio-monitoring Examination. Environmental Research, 109(6), 738–744. doi:10.1016/j.envres.2009.03.012
219. Srigboh, R. K., Basu, N., Stephens, J., Asampong, E., Perkins, M., Neitzel, R. L., & Fobil, J. (2016). Multiple elemental exposures amongst workers at the Agbogbloshie electronic waste (e-waste) site in Ghana. Chemosphere, 164, 68–74.doi:10.1016/j.chemosphere.2016.08.089

220. Takeda, S. H. K., Kuno, R., Barbosa, F., & Gouveia, N. (2017). Trace element levels in blood and associated factors in adults living in the metropolitan area of São Paulo, Brazil. Journal of Trace Elements in Medicine and Biology, 44, 307–314.doi:10.1016/j.jtemb.2017.09.005
221. Teeyapant P, Leudang S, Parnmen S. A cross-sectional study of exposure to mercury in schoolchildren living near the eastern seaboard industrial estate of Thailand. WHO South-East Asia J Public Health 2015;4:45-53
222. Tian, W., Egeland, G. M., Sobol, I., & Chan, H. M. (2011). Mercury hair concentrations and dietary exposure among Inuit preschool children in Nunavut, Canada. Environment International, 37(1), 42–48.doi:10.1016/j.envint.2010.05.017
223. Tomicic, C., Vernez, D., Belem, T., & Berode, M. (2011). Human mercury exposure associated with small-scale gold mining in Burkina Faso. International Archives of Occupational and Environmental Health, 84(5), 539–546. doi:10.1007/s00420-011-0615-x
224. Trasande, L., Cortes, J. E., Landrigan, P. J., Abercrombie, M. I., Bopp, R. F., & Cifuentes, E. (2010). Methylmercury exposure in a subsistence fishing community in Lake Chapala, Mexico: an ecological approach. Environmental Health, 9(1). doi:10.1186/1476-069x-9-1
225. UNUVAR, E., AHMADOV, H., KIZILER, A., AYDEMIR, B., TOPRAK, S., ULKER, V., & ARK, C. (2007). Mercury levels in cord blood and meconium of healthy newborns and venous blood of their mothers: Clinical, prospective cohort study. Science of The Total Environment, 374(1), 60–70.doi:10.1016/j.scitotenv.2006.11.043
226. Valera, B., Dewailly, E., & Poirier, P. (2011). Impact of mercury exposure on blood pressure and cardiac autonomic activity among Cree adults (James Bay, Quebec, Canada). Environmental Research, 111(8), 1265–1270.doi:10.1016/j.envres.2011.09.001
227. Valera, B., Dewailly, É., Poirier, P., Counil, E., & Suhas, E. (2011). Influence of mercury exposure on blood pressure, resting heart rate and heart rate variability in French Polynesians: a cross-sectional study. Environmental Health, 10(1). doi:10.1186/1476-069x-10-99
228. Vejrup, K., Brandlistuen, R. E., Brantsæter, A. L., Knutsen, H. K., Caspersen, I. H., Alexander, J., … Haugen, M. (2018). Prenatal mercury exposure, maternal seafood consumption and associations with child language at five years. Environment International, 110, 71–79. doi:10.1016/j.envint.2017.10.008
229. Vieira, H. C., Morgado, F., Soares, A. M. V. M., & Abreu, S. N. (2013). Mercury in Scalp Hair Near the Mid-Atlantic Ridge (MAR) in Relation to High Fish Consumption. Biological Trace Element Research, 156(1-3), 29–35.doi:10.1007/s12011-013-9849-7
230. Vigeh, M., Yokoyama, K., Ramezanzadeh, F., Dahaghin, M., Sakai, T., Morita, Y., … Kobayashi, Y. (2006). Lead and other trace metals in preeclampsia: A case–control study in Tehran, Iran. Environmental Research, 100(2), 268–275.doi:10.1016/j.envres.2005.05.005
231. Weil, M. (2005). Blood Mercury Levels and Neurobehavioral Function. JAMA, 293(15), 1875.doi:10.1001/jama.293.15.1875
232. Weinhouse, C., Ortiz, E., Berky, A., Bullins, P., Haregrogg, J., Rogers, L., ... & Pan, W. (2017). Hair Mercury Level is Associated with Anemia and Micronutrient Status in Children Living Near Artisanal and Small-Scale Gold Mining in the Peruvian Amazon.. American Journal of Tropical Medicine and Hygiene, 97(6), 1886-1897.
233. Wells, E. M., Jarrett, J. M., Lin, Y. H., Caldwell, K. L., Hibbeln, J. R., Apelberg, B. J., … Goldman, L. R.

(2011). Body burdens of mercury, lead, selenium and copper among Baltimore newborns. Environmental Research, 111(3), 411–417. doi:10.1016/j.envres.2010.12.009

234. Wijesekara, L. A., Usoof, R., Gamage, S. S. T., Jayasinghe, R., Gamage, N., De Silva, D., & Ekanayake, S. (2017). Mercury levels in hair samples of dentists: A comparative study in Sri Lanka. Journal of Investigative and Clinical Dentistry, 9(2), e12302.doi:10.1111/jicd.12302
235. Wittsiepe, J., Feldt, T., Till, H., Burchard, G., Wilhelm, M., & Fobil, J. N. (2016). Pilot study on the internal exposure to heavy metals of informal-level electronic waste workers in Agbogbloshie, Accra, Ghana. Environmental Science and Pollution Research, 24(3), 3097–3107.doi:10.1007/s11356-016-8002-5
236. Wu M, Yan C, Xu J, et al. Umbilical cord blood mercury levels in China[J]. Journal of Environmental Sciences-china, 2013, 25(2): 386-392.
237. Wyatt, L., Ortiz, E., Feingold, B., Berky, A., Diringer, S., Morales, A., … Pan, W. (2017). Spatial, Temporal, and Dietary Variables Associated with Elevated Mercury Exposure in Peruvian Riverine Communities Upstream and Downstream of Artisanal and Small-Scale Gold Mining. International Journal of Environmental Research and Public Health, 14(12), 1582.doi:10.3390/ijerph14121582
238. Xu, Y., Khoury, J. C., Sucharew, H., Dietrich, K., & Yolton, K. (2016). Low-level gestational exposure to mercury and maternal fish consumption: Associations with neurobehavior in early infancy. Neurotoxicology and Teratology, 54, 61–67.doi:10.1016/j.ntt.2016.02.002
239. Yard, E. E., Horton, J., Schier, J. G., Caldwell, K., Sanchez, C., Lewis, L., & Gastañaga, C. (2012). Mercury Exposure Among Artisanal Gold Miners in Madre de Dios, Peru: A Cross-sectional Study. Journal of Medical Toxicology, 8(4), 441–448.doi:10.1007/s13181-012-0252-0
240. Yard, E., Bayleyegn, T., Abebe, A., Mekonnen, A., Murphy, M., Caldwell, K. L., … Kebede, A. (2015). Metals Exposures of Residents Living Near the Akaki River in Addis Ababa, Ethiopia: A Cross-Sectional Study. Journal of Environmental and Public Health, 2015, 1–8. doi:10.1155/2015/935297
241. Ye, X., Qian, H., Xu, P., Zhu, L., Longnecker, M. P., & Fu, H. (2009). Nephrotoxicity, neurotoxicity, and mercury exposure among children with and without dental amalgam fillings. International Journal of Hygiene and Environmental Health, 212(4), 378–386.doi:10.1016/j.ijheh.2008.09.004
242. You, C.-H., Kim, B.-G., Jo, E.-M., Kim, G.-Y., Yu, B.-C., Hong, M.-G., … Hong, Y.-S. (2012). The relationship between the fish consumption and blood total/methyl-mercury concentration of costal area in Korea. NeuroToxicology, 33(4), 676–682.doi:10.1016/j.neuro.2012.04.005
243. You, C.-H., Kim, B.-G., Kim, Y.-M., Lee, S.-A., Kim, R.-B., Seo, J.-W., & Hong, Y.-S. (2014). Relationship between Dietary Mercury Intake and Blood Mercury Level in Korea. Journal of Korean Medical Science, 29(2), 176.doi:10.3346/jkms.2014.29.2.176
244. Zeng, Q., Feng, W., Zhou, B., Wang, Y.-X., He, X.-S., Yang, P., … Lu, W.-Q. (2015). Urinary Metal Concentrations in Relation to Semen Quality: A Cross-Sectional Study in China. Environmental Science & Technology, 49(8), 5052–5059.doi:10.1021/es5053478
245. Zubero, M. B., Aurrekoetxea, J. J., Ibarluzea, J. M., Arenaza, M. J., Rodríguez, C., & Sáenz, J. R. (2010). Heavy metal levels (Pb, Cd, Cr and Hg) in the adult general population near an urban solid waste incinerator. Science of The Total Environment, 408(20), 4468–4474.doi:10.1016/j.scitotenv.2010.07.003
