# Supplementary material for: Global health effects of future atmospheric mercury emissions
Source: Nat Commun. 2021 May 24;12:3035. doi: 10.1038/s41467-021-23391-7 (PMC8144432; doi:10.1038/s41467-021-23391-7)
Supplement: Supplementary file 3 — Reporting Summary [file 41467_2021_23391_MOESM3_ESM.pdf]

Corresponding author(s): Yanxu Zhang
Last updated by author(s): Apr 22, 2021

Reporting Summary

Nature Research wishes to improve the reproducibility of the work that we publish. This form provides structure for consistency and transparency in reporting. For further information on Nature Research policies, see our Editorial Policies and the Editorial Policy Checklist.

Statistics

For all statistical analyses, confirm that the following items are present in the figure legend, table legend, main text, or Methods section.

n/a	Confirmed	
☒	☐	The exact sample size (*n*) for each experimental group/condition, given as a discrete number and unit of measurement
☒	☐	A statement on whether measurements were taken from distinct samples or whether the same sample was measured repeatedly
☒	☐	The statistical test(s) used AND whether they are one- or two-sided<br>*Only common tests should be described solely by name; describe more complex techniques in the Methods section.*
☒	☐	A description of all covariates tested
☒	☐	A description of any assumptions or corrections, such as tests of normality and adjustment for multiple comparisons
☐	☒	A full description of the statistical parameters including central tendency (e.g. means) or other basic estimates (e.g. regression coefficient) AND variation (e.g. standard deviation) or associated estimates of uncertainty (e.g. confidence intervals)
☒	☐	For null hypothesis testing, the test statistic (e.g. *F*, *t*, *r*) with confidence intervals, effect sizes, degrees of freedom and *P* value noted<br>*Give P values as exact values whenever suitable.*
☒	☐	For Bayesian analysis, information on the choice of priors and Markov chain Monte Carlo settings
☒	☐	For hierarchical and complex designs, identification of the appropriate level for tests and full reporting of outcomes
☒	☐	Estimates of effect sizes (e.g. Cohen's *d*, Pearson's *r*), indicating how they were calculated

Our web collection on statistics for biologists contains articles on many of the points above.

Software and code

Policy information about availability of computer code

Data collection	We use Microsoft Excel 2019 for data collection.
Data analysis	We used Python 3.8 for data analysis. All model code is available at the research group website: https://www.ebmg.online/model_hg.html.

For manuscripts utilizing custom algorithms or software that are central to the research but not yet described in published literature, software must be made available to editors and reviewers. We strongly encourage code deposition in a community repository (e.g. GitHub). See the Nature Research guidelines for submitting code & software for further information.

Data

Policy information about availability of data

All manuscripts must include a data availability statement. This statement should provide the following information, where applicable:

- Accession codes, unique identifiers, or web links for publicly available datasets
- A list of figures that have associated raw data
- A description of any restrictions on data availability

All data is available in the Supplementary Information and the research group website: https://www.ebmg.online/model_hg.html. FAO/WHO global individual food consumption database: http://www.fao.org/nutrition/assessment/food-consumption-database/en/. World population prospects: https://population.un.org. Shared socioeconomic pathways database: https://tntcat.iiasa.ac.at/SspDb. Global hunger index: https://www.globalhungerindex.org. Fishbase database: https://www.fishbase.org. Marine trophic index: http://www.seaaroundus.org/mti-fib-rmti/. Global health estimates: http://www.who.int/healthinfo/global_burden_disease.

Field-specific reporting

Please select the one below that is the best fit for your research. If you are not sure, read the appropriate sections before making your selection.

☐ Life sciences ☐ Behavioural & social sciences ☒ Ecological, evolutionary & environmental sciences

For a reference copy of the document with all sections, see nature.com/documents/nr-reporting-summary-flat.pdf

Ecological, evolutionary & environmental sciences study design

All studies must disclose on these points even when the disclosure is negative.

Study description	We developed numerical models to simulate the environmental levels of mercury and its human health risk. We also predicted the response of the risk to projected future anthropogenic emissions.
Research sample	The study was based on numerical models and no sample analysis is conducted.
Sampling strategy	The study was based on numerical models and we did not perform any sampling.
Data collection	Y.Z., Z.S., S.H., P.Z., Y.P., P.W., and J.G. searched the public literature database and collected the reported food Hg data.
Timing and spatial scale	We collected all the available food Hg data in the literature ever reported for the global scale.
Data exclusions	We excluded the data points near point sources or contaminated sites.
Reproducibility	We repeated all the calculations once before submitting and it reproduced the results successfully.
Randomization	We used a Monte Carlo analysis to calculate the overall uncertainty of the calculated health risk.
Blinding	We calculated the mercury exposure health risk of the general population, which is not for any specific person. Therefore, no blinding protocol is required.

Did the study involve field work? ☐ Yes ☒ No

Reporting for specific materials, systems and methods

We require information from authors about some types of materials, experimental systems and methods used in many studies. Here, indicate whether each material, system or method listed is relevant to your study. If you are not sure if a list item applies to your research, read the appropriate section before selecting a response.

Materials & experimental systems

n/a	Involved in the study	
☒	☐	Antibodies
☒	☐	Eukaryotic cell lines
☒	☐	Palaeontology and archaeology
☒	☐	Animals and other organisms
☒	☐	Human research participants
☒	☐	Clinical data
☒	☐	Dual use research of concern

Methods

n/a	Involved in the study	
☒	☐	ChIP-seq
☒	☐	Flow cytometry
☒	☐	MRI-based neuroimaging
